# Biological Markers for Pulpal Inflammation: A Systematic Review

**DOI:** 10.1371/journal.pone.0167289

**Published:** 2016-11-29

**Authors:** Dan-Krister Rechenberg, Johnah C. Galicia, Ove A. Peters

**Affiliations:** 1 Department of Preventive Dentistry, Periodontology and Cariology, Center of Dental Medicine, University of Zürich, Zürich, Switzerland; 2 Department of Endodontics, Arthur A. Dugoni School of Dentistry, University of the Pacific, San Francisco, California, United States of America; Instituto Butantan, BRAZIL

## Abstract

**Background and Objective:**

Pulpitis is mainly caused by an opportunistic infection of the pulp space with commensal oral microorganisms. Depending on the state of inflammation, different treatment regimes are currently advocated. Predictable vital pulp therapy depends on accurate determination of the pulpal status that will allow repair to occur. The role of several players of the host response in pulpitis is well documented: cytokines, proteases, inflammatory mediators, growth factors, antimicrobial peptides and others contribute to pulpal defense mechanisms; these factors may serve as biomarkers that indicate the status of the pulp. Therefore, the aim of this systematic review was to evaluate the presence of biomarkers in pulpitis.

**Methods:**

The electronic databases of MEDLINE, EMBASE, Scopus and other sources were searched for English and non-English articles published through February 2015. Two independent reviewers extracted information regarding study design, tissue or analyte used, outcome measures, results and conclusions for each article. The quality of the included studies was assessed using a modification of the Newcastle-Ottawa-Scale.

**Results and Conclusions:**

From the initial 847 publications evaluated, a total of 57 articles were included in this review. In general, irreversible pulpitis was associated with different expression of various biomarkers compared to normal controls. These biomarkers were significantly expressed not only in pulp tissue, but also in gingival crevicular fluid that can be collected non-invasively, and in dentin fluid that can be analyzed without extirpating the entire pulpal tissue. Such data may then be used to accurately differentiate diseased from healthy pulp tissue. The interplay of pulpal biomarkers and their potential use for a more accurate and biologically based diagnostic tool in endodontics is envisaged.

## Introduction

**Fig 1:**
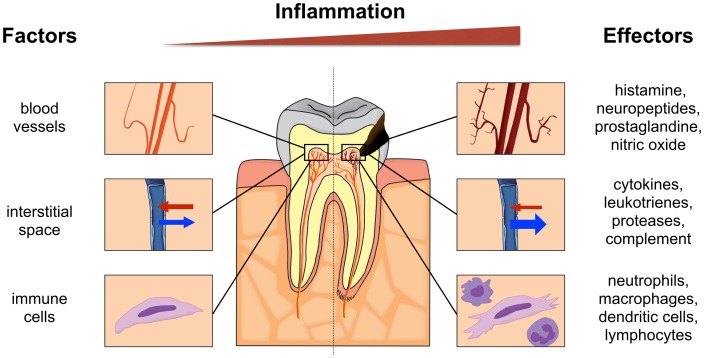
Schematic illustration of a tooth with a healthy pulp (left panel) and an inflamed pulp (right panel) subjacent to a caries lesion. Involved factors and biological effectors are indicated for both pulpal conditions.

The dental pulp is equipped to express numerous mediators of inflammation, which can combat irritating factors [1–4]. Its mechanistic response begins with vascular changes mediated by Toll-like receptors (TLR) 4/2-positive cells and includes release of measurable inflammatory mediators such as IL-8, IL-6, IL-1 and others [4–7]. Under normal physiologic conditions (left in Fig 1), the vasculature consists of central vessels that branch out into a plexus towards the periphery and specifically the pulp horns. An important difference from soft tissue-enclosed portions of the body is that dental hard tissues enclose the pulp creating a low compliance environment. Dental blood vessels are mainly under control by local metabolites and less by sympathetic innervation. The main cellular components of the pulp are peripherally located odontoblasts and stromal fibroblasts. There are also undifferentiated mesenchymal cells found mainly in the paravascular niche and immune cells (Fig 1). In health, neutrophils predominate but dendritic cells and occasional macrophages are also found.

Inflammation of the dental pulp (pulpitis) has been viewed as a tightly regulated sequence of vascular and cellular events mediated by molecular factors [8]. Pulpitis is typically caused by an opportunistic infection of the pulp space by commensal oral microorganisms [9]. The most common route of entry for the microorganisms is dental caries. Other potential pathways for pulpal microbial infection include trauma, dentinal cracks, exposed dentinal tubules or the main apical foramen [10]. Cells in human dental pulp that express TLR contribute trigger immune responses to microorganisms and their by-products [2–4]. This group includes odontoblasts [11], endothelial cells [12] as well as macrophages and dendritic cells [13]. Some of these cells may form mechanical barriers (i.e. odontoblasts), detect and transmit sensations (nerve fibers) or differentiate (i.e. dental pulp stem cells) to limit infection, signal injury and promote repair, respectively.

Based on the patients’ signs, symptoms, and examination, four clinical pulpal conditions are described: normal, reversibly inflamed, irreversibly inflamed or necrotic [14]. Histology represents the gold standard to determine the inflammatory state of pulp tissue [15, 16]; however, it is generally agreed that histologic and clinical classification of pulpal diagnosis still needs to be improved and refined. Normal and necrotic pulps have straightforward histological presentation. The conundrum lies in differentiating reversible and irreversible pulpitis. Based on histological reports, reversible pulpitis is characterized by the absence of bacteria and by localized coagulation and liquefaction necrosis immediately surrounding the irritant, whereas irreversible pulpitis is characterized by the presence of the bacteria or their by-products in the dental pulp and by preponderance of acute inflammatory cells predominantly neutrophils in the tissue beneath the lesion suggesting chemotactic activity. Lysosomal enzymes discharged by neutrophils result in widespread tissue damage and suppuration [16–18]. Acute pulpitis (reversible, and irreversible) can be an extremely painful condition and is believed to be one of the main causes for patients to seek emergency dental treatment during or after office hours [19, 20]. The main clinical difference between reversible and irreversible pulpitis is in the pulp’s response to thermal stimulus. Reversible pulpitis presents an exaggerated yet non-lingering response to cold stimulus. Irreversible pulpitis on the other hand is characterized by constant, spontaneous pain with exaggerated and lingering response to cold stimulus. However, forty percent of teeth with irreversible pulpitis can be painless [21]. In reversible pulpitis, the pulp is expected to recover after removal of the causative stimulus. In contrast, if the pulp is irreversibly inflamed, healing is not expected and pulpectomy (i.e., full removal of the dental pulp) is indicated.

The succession of signaling events resulting from dental pulp stimulation by microorganisms to the release of an array of immune mediators that in turn may cause pulpal or odontogenic pain, pulpitis, or in advanced stages, pulpal necrosis and finally apical periodontitis have been well described in the past [4–7]. Detailed discussion of these mechanisms is beyond the scope of this article.

Currently, diagnostic procedures that aim to assess pulpal inflammation involve case history, as well as clinical and radiographic examination. Clinical examination includes different procedures such as inspection, pulp sensitivity to thermal or electric stimuli, and pain on palpation or percussion. These procedures apparently did not change much in the last century [22]. However, the validity of the currently employed clinical tests to determine the actual or histopathological status of the pulp remains controversial [15]. A recently performed literature review summarized the available information on the diagnostic accuracy of signs/symptoms and current tests used to determine the condition of the pulp [23]. These authors concluded that the overall evidence was insufficient to support the accuracy of such test, even if the tests are combined. Hence, the current diagnostic procedures do not reliably identify the inflammatory status of the pulp. This is particularly unfortunate since decision making in this field, for example differentiation between vital pulp therapy and root canal treatment, critically depends on an accurate pulpal diagnosis.

According to the National Library of Medicines, the medical subject heading term (MeSH term) definition for a biological marker is a measurable and quantifiable biological parameter that serves as an indicator for health- and physiology-related assessments. Molecules expressed in the cascade of tissue inflammation may serve as (diagnostic) biomarkers for the presence of inflammation. Some research suggests that the dental pulp is not an isolated entity in an encased, solid environment but a reactive tissue that extends its biological products into the outside environment [24, 25]. In fact, studies have shown that pulpal events can be reflected through measurable levels of protein markers that correlated with pulpal symptoms in pulpal blood [26], dentinal fluid [27], periapical fluid [28], and gingival crevicular fluid (GCF; [1, 29]).

In the field of periodontology, biomarkers in oral fluids/saliva or gingival crevicular fluid are used to detect the occurrence and progression of periodontitis [30, 31]. For example, matrix metalloproteinases (MMPs) such as MMP-8 and -9 have been shown to be central biomarkers of soft tissue breakdown in periodontal pockets [32]. Periodontal and pulpal inflammation shares certain features: initially, both exhibit soft-tissue inflammation caused by microbial infection. At a later stage, these pathologic processes culminate in bone resorption (vertical bone-loss or apical periodontitis, respectively). It is therefore possible that both pathoses may express the same biomarkers. In this regard, MMPs were shown to be potential biomarker for both pulpal [33] and periodontal disease [32]. However, the application of molecular diagnostics in pulpal disease is as yet not used for clinical decision-making [34].

Previous studies have investigated the molecular regulatory pathways of pulpal inflammation employing explanted cell cultures *in vitro* [35–37]. However, the extrapolation of such results to the clinical situation is difficult, perhaps due to the reductionist nature of such experiments. *In vivo*, the presence of other cellular players (e.g. immune cells), inhibitory proteins (e.g. protease inhibitors) and other molecules that modify the inflammatory response may present a completely different inflammatory response and consequently, a different clinical outcome compared with what may be suggested by *in vitro* experimental results. Studies reporting clinical samples for the presence of potential biomarkers for pulpal inflammation are still on the rise. The clinical importance of identifying these biomarkers that can be used to diagnose or to stage pulpal inflammation warrants not only additional studies but also a critical or systematic review and analysis of published reports. Therefore, the aim of this paper is to systematically review the currently available information on biomarkers that were identified from pulp tissues diagnosed as normal or inflamed.

## Systematic Review

### Eligibility Criteria and Literature Search

This systematic review was prepared in accordance with PRISMA (S1 Table) [38]. Studies were eligible for inclusion to the review that clinically and/ or histologically differentiate between a healthy and a irreversibly inflamed pulp in permanent human teeth, and analyzed interstitial/ dentinal liquor, gingival crevicular fluid, pulpal tissue, dentin fluid or apical blood for the presence of a biological marker. A biological marker is defined as measurable and quantifiable biological molecule that theoretically can be present in those substrates and might serve as an indicator for a healthy or diseased pulp (adapted from MeSH Unique ID: D015415). An electronic search strategy with combined keywords and indexing vocabulary (MeSH terms) was conducted in the Medline database of the US National Library of Medicine employing the OvidSP interface. We used the following search terms and other subject headings: ‘pulpitis’, ‘acute pulpitis’, ‘irreversible pulpitis’, ‘painful pulpitis’, ‘biological markers’, ‘inflammation mediators’, ‘dentinal fluid’, and ‘gingival crevicular fluid’. S2 Table lists the detailed search strategy performed in Medline. The same electronic search strategy was used in Biosis (OvidSP), the Cochrane library (Wiley), Embase (http://www.embase.com) and the Web of Science (Thomson Reuters). The last date entered was February 19, 2015. No language restrictions were imposed and all articles were included from the inception of the respective database (S3 Table). To ensure the completeness of the search, one reviewer (DRK) conducted a thorough search of the bibliographies of all included studies.

### Study Selection and Quality Assessment

**Fig 2:**
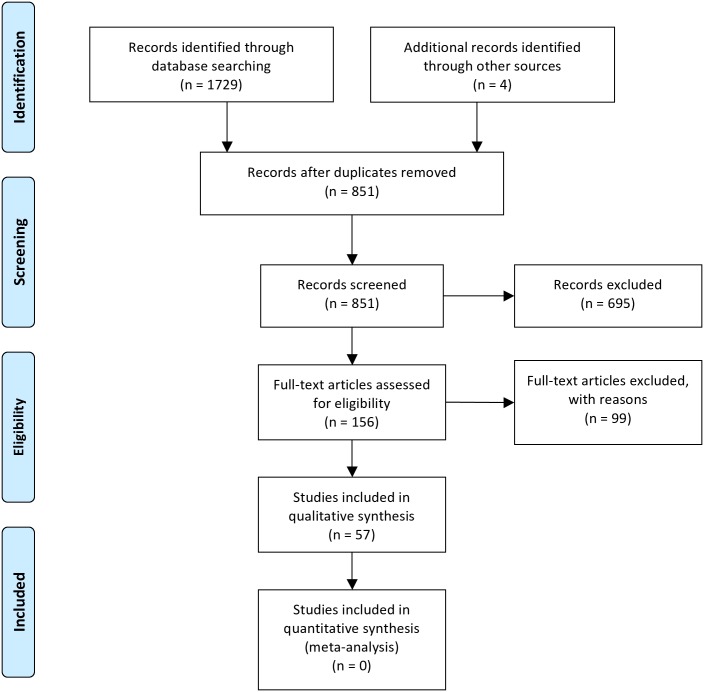
PRISMA flowchart depicting the systematic selection and exclusion of articles related to the topic. A detailed description of the excluded articles with the respective reasons for exclusion is presented in the running text and S4 Table. *From*: Moher D, Liberati A, Tetzlaff J, Altman DG, The PRISMA Group (2009). *P*referred *R*eporting *I*terns for Systematic Reviews and *M*eta-*A*nalyses: The PRISMA Statement. PLoS Med 6(7): e1000097. doi: 10.1371/journal.pmed1000097 **For more information, visit** www.prisma-statement.org.

The search and selection process is summarized in Fig 2 [38]. A pool of 1733 records was initially identified using the electronic search strategy and other sources. After removal of duplicates, 851 records remained. Two reviewers (DKR and JCG) independently screened the titles and abstracts of the references collected. Communications not related to the topic were discarded (n = 695). Communications deemed appropriate by one of the reviewers were assigned for full text evaluation. One hundred and fifty-six records were identified using this approach and reviewed as full texts. Articles were collected and evaluated independently by both reviewers. Non-English abstracts or manuscripts were translated with the help of translators. Further articles (n = 99) were excluded for one of the following reasons: i) studies not on human teeth, ii) cell culture study only, iii) no potential biomarker was investigated or the study was off topic, iv) no clear distinction between reversible, irreversible or necrotic pulp, v) studies rather on histologic features or presence of cells, bacteria or viruses than on quantification of a biomarker, vi) review articles, editorials, comments, abstract only or case reports (S4 Table). In case of disagreement consensus was achieved through discussion by third party arbitration (OAP). Articles where no exclusion criteria applied were included to the review. There was 94.2% agreement prior to arbitration between both reviewers and finally 57 publications were included to the review. The included articles were written in English (n = 54) or Chinese (n = 3) language.

### Quality Assessment

The quality of the included studies was assessed using a modification of the Newcastle-Ottawa-Scale (NOS; [39, 40]). The NOS rates the 3 study domains ‘selection’, ‘comparability’ and ‘outcome’. Each positive rating was awarded with a star. The parameters recorded for ‘selection’ were: selection of the cohort (gender and age distribution reported) and condition of the cohort (general health and medication reported). The parameters recorded for ‘comparability’ were: diagnostics of cases and controls (anamnesis, clinical and radiological inspection described in sufficient detail), histological confirmation of the diagnosis performed (yes/no), quality of the controls (control sample from the same patient as the case sample) and the ratio of the group size (cases:controls ≥ 1:2). The parameters recorded for ‘outcome’ were: reported blinding to the case/control status (yes/no) and that the same tests were performed with the cases and control samples (yes/no). Consequently two stars could be awarded for ‘selection’, four stars for ‘comparability’ and two stars for ‘outcome’. A value of ‘0’ represents the lowest study quality and ‘8’ the highest possible quality rating of the modified NOS.

### Data Extraction and Statistical Analysis

Quantitative data were collected from all studies included to the review. An electronic protocol for data extraction was defined and piloted on several manuscripts before final completion. Relevant information regarding reference name, publication date, substrate analyzed for the presence of a biological marker, how was the substrate collected, number of specimens in experimental group and control group, was the substrate pooled before analysis (yes/no), name of the biological marker under investigation, what type of molecule is the biological marker, what general function serves the biological marker, what was the molecular expression level of the biological marker, which analyte was evaluated for the presence of the biological marker, which method was used for analysis, and were statistically significant differences between specimens of the irreversible pulpitis group compared to the control group (healthy pulp) reported (yes/no), were collected. The synthesis of the data is presented in a descriptive manner. Moreover, descriptive statistics were applied when deemed appropriate (JMP 10.0.0, SAS Institute, Cary, N.C., USA).

## Results

### Study Characteristics and Quality Assessment

**Fig 3:**
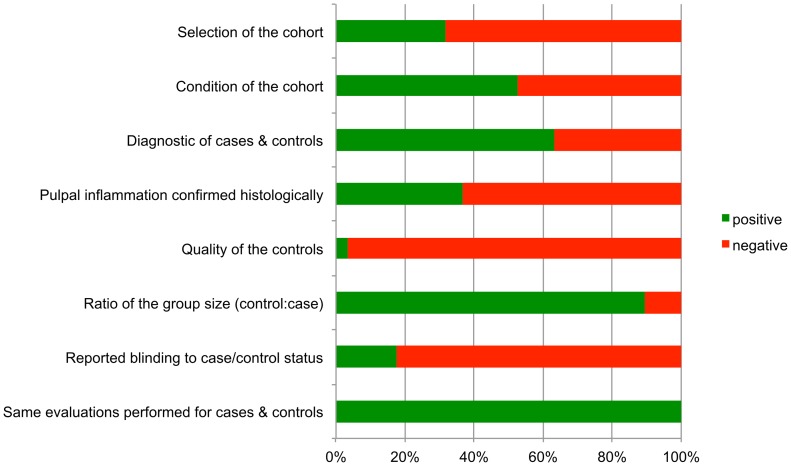
Bar chart showing the quality ratings of the included studies based on a modified Newcastle-Ottawa-Scale.

The studies excluded during full text evaluation (N = 99; Fig 2) are presented in S4 Table. The studies included in the review are listed in Tables 1 and 2. Due to the heterogeneous nature of the studies it was not possible to perform meta-analysis on their outcome. The quality ratings of the included studies according to the modified NOS are presented, along with the full reference, in S5 Table. The average quality score was 3.9 ± 1.1 (mean ± SD). Fig 3 provides an overview on the total ratings for the respective parameters for the study domains selection, comparability and outcome. Weaknesses were noticed for the parameters selection of the cohort, quality of the controls and reported blinding to the case/control status.

**Table 1:** Studies assessing pulp tissue for the presence of biomarkers associated with pulpal condition.

Reference	*n* per group (irreversibly inflamed/ non-inflamed)	Biomarker	Function	Target	Analyte	Method	Significant difference between groups
**Cytokines**							
Zehnder et al. 2003 [48]	11/13	IL-1α	Regulates immune and inflammatory reactions; stimulates bone resorption	mRNA	PTS	RT-PCR	n
Abd-Elmeguid et al. 2013* [49]	12/30			Protein	PTS	Multiplex assay	y
Abd-Elmeguid et al. 2013* [49]	12/30	IL-1rα	Acute phase protein that increases neutrophil presence of	Protein	PTS	Multiplex assay	y
Zehnder et al. 2003 [48]	11/13	IL-1β	Regulates immune and inflammatory reactions; stimulates bone resorption	mRNA	PTS	RT-PCR	n
Paris et al. 2009 [50]	10/7			mRNA	PTS	RT-PCR	y
Silva et al. 2009* [51]	5/5			Protein	PTS	ELISA	n
Abd-Elmeguid et al. 2013* [49]	12/30			Protein	PTS	Multiplex assay	y
Rauschenberger et al. 1997* [52]	15/17	IL-2	Regulates activities of leukocytes	Protein	PTS	ELISA	y
Anderson et al. 2002 [53]	32/24			Protein	PTS	ELISA	n
Abd-Elmeguid et al. 2013* [49]	12/30	IL-4	Key regulator in humoral and adaptive immunity; stimulates activated B cells, T-cell proliferation, and the differentiation of B-cells into plasma cells	Protein	PTS	Multiplex assay	y
Zehnder et al. 2003 [48]	11/13	IL-6	Regulator of T- and B-cell growth, acute phase protein production	mRNA	PTS	RT-PCR	y
Abd-Elmeguid et al. 2013* [49]	12/30			Protein	PTS	Multiplex assay	y
Abd-Elmeguid et al. 2013* [49]	12/30	IL-7	Stimulates proliferation and maturation of B and T cells	Protein	PTS	Multiplex assay	y
Huang et al. 1999* [54]	14/15	IL-8	Recruitment and activation of neutrophils	Protein	PTS	ELISA	y
Zehnder et al. 2003 [48]	11/13			mRNA	PTS	RT-PCR	y
Silva et al. 2009* [51] *	5/5			Protein	PTS	ELISA	n
Abd-Elmeguid et al. 2013* [49]	12/30			Protein	PTS	Multiplex assay	y
Abd-Elmeguid et al. 2013* [49]	12/30	IL-12p40	Subunit of IL-12; acts on T- and natural killer cells	Protein	PTS	Multiplex assay	y
Abd-Elmeguid et al. 2013* [49]	12/30	IL-13	Mediator of allergic inflammation and disease	Protein	PTS	Multiplex assay	y
Abd-Elmeguid et al. 2013* [49]	12/30	IL-15	Induces proliferation of natural killer cells	Protein	PTS	Multiplex assay	n
Zehnder et al. 2003 [48]	11/13	IL-18	Pro-inflammatory cytokine involved in cell mediated immunity	mRNA	PTS	RT-PCR	y
Pezelj-Ribaric et al. 2002 [55]	19/18	TNF-α	Delays neutrophil apoptosis	Protein	PTS	ELISA	y
Kokkas et al. 2007 [43]	6/6			mRNA	PTS	RT-PCR	y
Keller et al. 2009 [56]	5/5			mRNA	PTS	RT-PCR	y
Paris et al. 2009 [50]	10/7			mRNA	PTS	RT-PCR	y
Abd-Elmeguid et al. 2013* [49]	12/30			Protein	PTS	Multiplex assay	y
Abd-Elmeguid et al. 2013* [49]	12/30	TNF-β	Mediates a large variety of inflammatory, immunostimulatory, and antiviral responses	Protein	PTS	Multiplex assay	n
Li et al. 2011* [57]	4/4	MIP-1α	Mediate immune responses towards infection and inflammation; activation of granulocytes	mRNA	PTS	RT-PCR	y
Abd-Elmeguid et al. 2013* [49]	12/30			Protein	PTS	Multiplex assay	y
Abd-Elmeguid et al. 2013* [49]	12/30	MIP-1β	Mediate immune responses towards infection and inflammation; activation of granulocytes	Protein	PTS	Multiplex assay	y
Nakanishi et al. 2005* [58]	8/5	MIP-3α	Chemoattractant for lymphocytes and neutrophils	Protein	FPT	IHC	n/a
Nakanishi et al. 2005* [58]	8/5	CCR6	MIP-3α Receptor on memory T-cells, dendritic cells and Th17 cells	Protein	FPT	IHC	n/a
Abd-Elmeguid et al. 2013* [49]	12/30	TGF-α	Induces epithelial development and wound healing	Protein	PTS	Multiplex assay	y
Piattelli et al. 2004* [59]	20/23	TGF-β1	Modulates pro-inflammatory cytokine production, inhibits mitogenic effects of IL-2 on T and B lymphocytes, blocks activity of other immunocompetent cells	Protein	FPT	IHC	y
Adachi et al. 2007* [60]	9/4	CXCL10	Chemoattractant for monocytes/macrophages, T cells, NK cells, and dendritic cells	Protein	PTS	RT-PCR	y
	5/4			Protein	FPT	IHC	n/a
Jiang et al. 2008* [61]	6/5	SDF-1	Chemotactic for lymphocytes	mRNA	PTS	RT-PCR	y
	4/4			Protein	FPT	IHC	n/a
Huang et al. 2009* [62]	15/15	Oncostatin M	Involved in hematopoiesis, tissue remodelling processes and inflammation	mRNA	PTS	RT-PCR	y
				Protein	FPT	IHC	y
Abd-Elmeguid et al. 2013* [49]	12/30	GM-CSF	Stimulates production of granulocytes and monocytes	Protein	PTS	Multiplex assay	y
Abd-Elmeguid et al. 2013* [49]		GRO	Neutrophil chemoattractant. Involved in angiogenesis, inflammation, wound healing and tumorigenesis	Protein	PTS	Multiplex assay	y
Abd-Elmeguid et al. 2013* [49]		MCP-1	Chemoattractant for monocytes, recruits memory T cells, and dendritic cells to the sites of inflammation	Protein	PTS	Multiplex assay	y
Abd-Elmeguid et al. 2013* [49]		MCP-3	Chemoattractant for monocytes; regulates macrophage function	Protein	PTS	Multiplex assay	y
Abd-Elmeguid et al. 2013* [49]		MDC	Chemotactic for monocytes, dendritic cells and natural killer cells	Protein	PTS	Multiplex assay	y
Abd-Elmeguid et al. 2013* [49]		INF-α	Antiviral agents, modulate functions of the immune system	Protein	PTS	Multiplex assay	y
Abd-Elmeguid et al. 2013* [49]		G-CSF	Stimulates proliferation and differentiation of granulocytes	Protein	PTS	Multiplex assay	y
Abd-Elmeguid et al. 2013* [49]		Eotaxin-1	Recruits eosinophils by inducing their chemotaxis	Protein	PTS	Multiplex assay	y
Abd-Elmeguid et al. 2013* [49]		flt3ligand	Stimulates proliferation and differentiation of various blood cell progenitors	Protein	PTS	Multiplex assay	y
Abd-Elmeguid et al. 2013* [49]		Fractalkine	Chemoattractant for T cells and monocytes; promotes strong adhesion of leukocytes to activated endothelial cells	Protein	PTS	Multiplex assay	y
Abd-Elmeguid et al. 2013* [49]		CD40L	Co-stimulatory molecule for T cells; promotes B cell maturation and function	Protein	PTS	Multiplex assay	n
Abd-Elmeguid et al. 2013* [49]		sIL-2rα	Receptor that mediates IL-2 activities; increased levels biological fluids correlate with increased immune system activation	Protein	PTS	Multiplex assay	n
Abd-Elmeguid et al. 2013* [49]		IP-10	Chemoattractant for monocytes/macrophages, T cells, NK cells, and dendritic cells	Protein	PTS	Multiplex assay	n
Abd-Elmeguid et al. 2013* [49]		PDGF-AA	Receptor that regulates cell proliferation, cellular differentiation, cell growth and development	Protein	PTS	Multiplex assay	n
Abd-Elmeguid et al. 2013* [49]		PDGF-AB/BB	Receptor that regulates cell proliferation, cellular differentiation, cell growth and development	Protein	PTS	Multiplex assay	n
Abd-Elmeguid et al. 2013* [49]		RANTES	Chemoattractant for leukocytes to inflammatory sites; proliferation and activation of natural-killer cells	Protein	PTS	Multiplex assay	n
Abd-Elmeguid et al. 2013* [49]		Osteocalcin	Regulation of bone mineralization	Protein	PTS	Multiplex assay	y
				Protein	FPT	IHC	n/a
**Proteases and other enzymes**							
Gusman et al. 2002 [63]	17/18	MMP-1	Regulator of connective tissue remodeling	Protein	PTS	ELISA	*Not detected*
Gusman et al. 2002 [63]	17/18	MMP-2	Hydrolysis of intercellular matrix	Protein	PTS	ELISA	y
Accorsi-Mendonca et al. 2013 [64]	10/10			Protein	PTS	Zymography	y
Accorsi-Mendonca et al. 2013 [64]	10/10	pro-MMP-2	Pro-form of MMP-2	Protein	PTS	Zymography	n
Gusman et al. 2002 [63]	17/18	MMP-3	Hydrolysis of intercellular matrix	Protein	PTS	ELISA	y
Tsai et al. 2005* [65]	14/14			mRNA	PTS	RT-PCR	y
				Protein	FPT	IHC	y
Gusman et al. 2002 [63]	17/18	MMP-9	Hydrolysis of intercellular matrix; regulatory factor for neutrophil migration across basement membrane	Protein	PTS	ELISA	y
Suwanchai et al. 2012 [66]	7/18			Protein	PTS	Western Blot	y
Accorsi-Mendonca et al. 2013 [64]	10/10			Protein	PTS	Zymography	n/a
Huang et al. 2005* [67]	17/13	t-PA	Involved in soft-tissue breakdown; catalyzes the conversion of plasminogen to plasmin	mRNA	PTS	RT-PCR	y
				Protein	FPT	IHC	n/a
Huang et al. 2007* [68]	22/9			Protein	PTS	Zymography	y
	22/9			Protein	PTS	ELISA	y
Ge et al. 1996 [69]	12/9	SOD	Antioxidant	Protein activity	PTS	Enzyme assay	y
Tulunoglu et al. 1998 [70]	10/7			Protein activity	PTS	Enzyme assay	n
Bodor et al. 2007 [71]	16/10	Cu, ZN-SOD	Protection against reactive oxygen species	mRNA	PTS	RT-PCR	y
Varvara et al. 2005 [72]	13/12			Protein activity	PTS	Enzyme assay	y
Bodor et al. 2007 [71]	16/10	Mn-SOD	Protection against reactive oxygen species	mRNA	PTS	RT-PCR	y
Ge et al. 1996 [69]	12/9	MDA	Oxidative stressor	Protein activity	PTS	Enzyme assay	y
Cootauco et al. 1993* [73]	5/8	Elastase	Cleavage of elastin, collagen, proteoglycans	Protein	FPT	IHC	y
		Cathepsin-G	Proteolysis	Protein	FPT	IHC	y
Spoto, Fioroni, Rubini, Tripodi, Di Stilio, et al. 2001 [74]	10/10	Alkaline phosphatase	Hydrolysis of phosphate ester-bonds	Protein activity	PTS	Enzyme assay	n
Spoto, Fioroni, Rubini, Tripodi, Perinetti, et al. 2001 [75]	20/20	Aspartate Aminotransferase	Catalyzes transfer of aminotransferase amino group of aspartate to α-ketoglutarat	Protein activity	PTS	Enzyme assay	n
Esposito, Varvara, Caputi, et al. 2003 [76]	15/18	Catalase	Catalyzes the breakdown of hydrogen peroxide	Protein activity	PTS	Enzyme assay	y
Esposito, Varvara, Murmura, et al. 2003 [77]	12/11			Protein activity	PTS	Enzyme assay	y
da Silva et al. 2008* [78]	6/6	NADPH-diaphorase	Detoxification to produce ROS	Protein	FPT	IHC	y
Di Nardo Di Maio et al. 2004* [79]	10/10	eNOS	Nitric oxide synthase	mRNA	PTS	RT-PCR	y
				Protein	PTS	Western blot	y
				Protein	FPT	IHC	y
	10/10	iNOS	Nitric oxide synthase	mRNA	PTS	RT-PCR	y
				Protein	PTS	Western blot	y
				Protein	FPT	IHC	y
Spoto, Ferrante, et al. 2004 [80]	6/12	cGMP PDE	Hydrolysis of cyclic nucleotide	Protein activity	PTS	Enzyme assay	y
Spoto, Menna, et al. 2004 [81]	6/12	cAMP PDE	Hydrolysis of cyclic nucleotide	Protein activity	PTS	Enzyme assay	y
Accorsi-Mendonca et al. 2013 [64]	10/10	TIMP-2	Inhibits MMP-2	Protein	PTS	ELISA	y
		MPO	Generation of reactive oxygen species	Protein activity	PTS	Enzyme assay	y
**Inflammatory mediators**							
Bolanos and Seltzer 1981 [82]	17/7	cAMP	Activation of protein kinases	Protein	PTS	RIA	n
		cGMP	Activation of protein kinases	Protein	PTS	RIA	n
Cohen et al. 1985 [47]	13/20	PGE2	Multiple pro-inflammatory and immunomodulatory effects	Protein	PTS^†^	RIA	y
		PGF2α	Multiple pro-inflammatory and immunomodulatory effects	Protein	PTS^†^	RIA	y
Cootauco et al. 1993* [73]	5/8	α-2M	Neutralization of proteinases	Protein		IHC	n/a
Dong et al. 1999 [83]	9/11	6-K-PGF1α	Vasodilators; inhibits the aggregation of blood platelets; involved in inflammation	Protein	PTS	RIA	y
		TXB2	Involved in platelet aggregation, vasoconstriction and reproductive functions	Protein	PTS	RIA	y
Khabbaz et al. 2001 [84]	15/5	Endotoxins	Induces strong immune response	Protein activity	PTS	LAL	y
Nakanishi et al. 2001* [85]	10/5	COX-2	Prostaglandin synthesis	Protein		IHC	n/a
Guven et al. 2007* [86]	12/12			Protein	FPT	IHC	n/a
Awawdeh et al. 2002 [87]	46/20	Substance P	Vasoactive mediator, immune mediator	Protein	PTS	RIA	y
Caviedes-Bucheli et al. 2006 [88]	6/6			Protein	PTS	RIA	y
Awawdeh et al. 2002 [87]	46/20	Neurokinin A	Generates three different preprotachykinins	Protein	PTS	RIA	y
Caviedes-Bucheli et al. 2006 [88]	6/6			Protein	PTS	RIA	y
Awawdeh et al. 2002 [87]	46/20	CGRP	Vasodilation and increased microvascular permeability	Protein	PTS	RIA	y
Caviedes-Bucheli et al. 2004 [89]	5/5			Protein	Pulp cells in suspension	Flow cytometry	y
Caviedes-Bucheli et al. 2005 [90]	6/4			Protein	PTS	RIA	y
Caviedes-Bucheli et al. 2006 [88]	6/6			Protein	PTS	RIA	y
Caviedes-Bucheli et al. 2006 [88]	6/6	Neuro-peptide Y	Potent vasoconstrictor, parasympathetic nervous system	Protein	PTS	RIA	y
		VIP	Vasodilator, parasympathetic nervous system	Protein	PTS	RIA	n
da Silva et al. 2008* [78]	6/6	NOD2	Involved in host response against bacteria	mRNA	PTS	RT-PCR	y
Keller et al. 2009 [56]	5/5			mRNA	PTS	RT-PCR	y
**Growth Factors**							
Artese et al. 2002* [91]	25/25	VEGF	Stimulates vasculogenesis and angiogenesis	Protein	FPT	IHC	n/a
Guven et al. 2007* [86]*	12/12			Protein	FPT	IHC	n/a
Abd-Elmeguid et al. 2013* [49]	12/30			Protein	PTS	Multiplex assay	n
Abd-Elmeguid et al. 2013* [49]	12/30	FGF	Involved in angiogenesis, wound healing, embryonic development and various endocrine signaling pathways	Protein	PTS	Multiplex assay	n
**Antimicrobial peptides**							
Paris et al. 2009 [50]	10/7	hBD-1	Activates the innate and adaptive immune system. Chemotactic for monocytes, T-lymphocytes, dendritic cells and mast cells	mRNA	PTS	RT-PCR	y
		hBD-2		mRNA	PTS	RT-PCR	n
		hBD-3		mRNA	PTS	RT-PCR	n
		hBD-4		mRNA	PTS	RT-PCR	y
**Others**							
Caviedes-Bucheli et al. 2007 [92]	5/5	Substance P receptor	Vasoactive mediator, immune mediator	Protein	PTS	RIA	y
Caviedes-Bucheli, Moreno, et al. 2008 [93]	13/13	AAMø CD163+ expressing CGRPr	Alternatively activated polarized monocyte/macrophage; different phenotype compared to the classical ones. Then expressing CD163+	Protein	Pulp cells in suspension	Flow cytometry	*See text*
Suwanchai et al. 2012 [66]	7/18	NaV 1.8	Initiation and propagation of action potentials; involved in pain perception	Protein	PTS	Western blot	y
		NaV 1.9	Initiation and propagation of action potentials; involved in pain perception	Protein	PTS	Western blot	y
Zhong et al. 2012 [94]	18/12	miRNAs	Regulators of post-transcriptional gene expression in biological processes like inflammation, immune response, and osteoclastic bone resorption	mRNA	PTS	Microarray	*See text*
Dong et al. 2013* [95]*	21/12	EphA7	Involved in embryonic development, angiogenesis, tumorigenesis, inflammation & pain	Protein	FPT	IHC	y
				mRNA	PTS	RT-PCR	y

* Pulpal inflammation confirmed histologically;

^†^ Substrate pooled before analysis;

y: Yes; n: No; n/a: Not applicable.

Analytes were mostly either pulp tissue supernatant (PTS) or fixed pulp tissue (FPT). One study used pulp cells in suspension, another one pulpal fluid. Analytical methods used included reverse transcription polymerase chain reaction (RT-PCR), multiplex assay, microarray, Western Blot, radioimmunoassay (RIA), immunohistochemistry (IHC), enzyme-linked immunosorbent assay (ELISA), zymography, flow cytometry, limulus amoebocyte assay (LAL), and specific enzyme assays.

**Table 2:** Studies assessing other substrates than pulp tissue for the presence of a biomarker.

Reference	*n* in group (irreversibly inflamed/ non-inflamed)	Biomarker	Function	Target	Analyte	Analysis	Significant difference between groups
**Cytokines**							
Nakanishi et al. 1995 [44]	27/9	IL-1α	Regulates immune and inflammatory reactions; stimulates bone resorption	Protein	PB	ELISA	n
		IL-1β	Regulates immune and inflammatory reactions; stimulates bone resorption	Protein	PB	ELISA	n
Elsalhy et al. 2013 [45]	43/25	IL-2	Regulates the activities of leukocytes	Protein	PB	ELISA	n
Nakanishi et al. 1995 [44]	27/9	IL-6	Regulator of T- and B-cell growth, acute phase protein production	Protein	PB	ELISA	*Not detected*
Elsalhy et al. 2013 [45]	43/25			Protein	PB	ELISA	y
Karapanou et al. 2008 [1]	17/17	IL-8	Recruitment and activation of neutrophils	Protein	GCF	ELISA	y
Elsalhy et al. 2013 [45]	43/25			Protein	PB	ELISA	y
Elsalhy et al. 2013 [45]	43/25	IL-10	Multiple effects in immunoregulation and inflammation; anti-inflammatory	Protein	PB	ELISA	y
Nakanishi et al. 1995 [44]	27/9	TNF-α	Delays neutrophil apoptosis	Protein	PB	ELISA	n
Karapanou et al. 2008 [1]	25/25			Protein	GCF	ELISA	*Not detected*
Elsalhy et al. 2013 [45]	43/25			Protein	PB	ELISA	y
Elsalhy et al. 2013 [45]	43/25	IFN-γ	Cytokine that is critical for innate and adaptive immunity	Protein	PB	ELISA	y
**Proteases and other enzymes**							
Zehnder et al. 2011 [33]	16/12	MMP-9	Hydrolysis of intercellular matrix; regulatory factor for neutrophil migration across basement membranes	Protein activity	Dentinal fluid	Enzyme assay	y
Nakanishi et al. 1995 [44]	27/9	Elastase	Cleavage of elastin, collagen, proteoglycans	Protein	PB	ELISA	y
**Vasoactive agents**							
Lepinski et al. 2000 [41]	11/10	Bradykinin	Vasodilator involved in pain and inflammation mechanisms	Protein	Extracellular pulpal fluid	RIA	y
Bowles et al. 2003 [42]	16/8			Protein	Extracellular pulpal fluid	RIA	y
**Others**							
Nakanishi et al. 1995 [44]	27/9	IgG	Antigen neutralization	Protein	PB	ELISA	y
Nakanishi et al. 1995 [44]	27/9	IgA	Antigen neutralization	Protein	PB	ELISA	y
Nakanishi et al. 1995 [44]	27/9	IgM	Antigen neutralization	Protein	PB	ELISA	y
Nakanishi et al. 1995 [44]	27/9	PGE2	Multiple pro-inflammatory and immunomodulatory effects	Protein	PB	ELISA	y
Evcil et al. 2006 [46]	16/16	Serum NO	Cellular signaling molecule involved in many physiological and pathological processes	Protein activity	Peripheral blood serum	Serum assay	n

y: Yes; n: No.

Analytes were mostly either pulpal blood (PB) or gingival crevicular fluid (GCF). Extracellular pulpal fluid and peripheral serum were used in one study each. Analytical methods used included radioimmunoassay (RIA), enzyme-linked immunosorbent assay (ELISA), and specific serum or enzyme assays.

### Tissues Studied

Eighty-eight percent of the studies included (50/57; Table 1) analyzed pulp tissue for the presence of a biomarker either collected via pulpectomy (N = 5), tooth extraction and fracturing (N = 25), or a combination of both (N = 20). Twelve percent (7/57; Table 2) of the studies included analyzed substrates other than pulp tissue: pulpal blood (N = 2), peripheral blood serum (N = 1), GCF (N = 1), dentinal fluid (N = 1), or extracellular pulpal fluid (N = 2). Pulpal blood, GCF and dentinal fluid were collected using absorbable membranes, blood serum via peripheral blood collection and extracellular pulpal fluid by inserting microdialysis membranes into vital pulp tissue [41, 42]. Eighty-two percent of the studies analyzing pulp tissue (41/50) used tissue collected from extracted healthy, non-carious permanent, or wisdom teeth as their control. Fourteen percent (7/50) used healthy pulp tissue collected via pulpectomy because of elected root canal treatment for prosthetic reasons as their control. One study used tissues from extraction and pulpectomy as control [43], another one did not state precisely how they collected control tissue [2]. Of the 7 studies evaluating substrates other than pulp tissue, two sampled blood [44, 45], and another one extracellular fluid [41] from healthy teeth that were assessed but subsequently planed for extraction because of prosthetic or orthodontic reasons as control. One study sampled venous (peripheral) blood during pulp inflammation and used a consecutive peripheral blood sample after treatment as control [46]. One further study sampled GCF from healthy contralateral or adjacent teeth as control [1], and another one collected dentinal fluid from non-symptomatic teeth scheduled for replacement of a filling as control [33]. The substrate in one study was pooled before performing the confirmatory test [47].

### Confirmatory Tests

Analytical methods used for the assessment of pulp tissue included reverse transcription polymerase chain reaction, multiplex assay, microarray, western blot, radioimmunoassay, immunohistochemistry, enzyme-linked immunosorbent assay, zymography, flow cytometry, limulus amoebocyte assay and specific enzyme assays (Table 1). Pulpal inflammation was confirmed by histology in 42% (21/50; Table 1 and S5 Table) of these studies. Substrates other than pulp tissue were analyzed using radioimmunoassay, enzyme-linked immunosorbent assay, specific serum, or enzyme assays (Table 2). Histology was not used to confirm pulpal diagnosis in those studies. Seventy-four percent of the studies evaluating pulp tissue (37/50) analyzed actual protein expression or protein activity, whereas 16% (8/50) analyzed the pulp tissue on the DNA level. Five studies (10%) analyzed the pulp tissue substrates at both levels (Table 1). All studies evaluating other substrates than pulp tissue evaluated protein expression or protein activity (Table 2).

### Markers Studied

Pulp tissue was assessed for a total of 89 biological markers. Statistical significant differences between an irreversible inflamed and a healthy pulp could be detected for 64 biological markers (71.9%) by at least one study. Nineteen biological markers showed no statistically significant differences between inflammation and health, whereas 6 biological markers were not evaluated employing statistical tests (Table 1). Substrate other than pulp tissue was evaluated for 16 biological markers. For twelve biological marker (75%) statistical significant differences between irreversible inflammation and health could be detect by at least one study.

## Discussion

The results point to a response in pulpitis by immunocompetent tissues that ultimately results in the release of mediators, which in turn trigger a series of inflammatory events and an attempt to initiate repair. Collectively, the data presented here demonstrate the involvement of various TLR-induced chemotactic molecules (i.e. IL-8, CXCL-10, MIP family, GRO, MCP family, RANTES, Eotaxin, IP10, and others).

TLRs have been shown to confer immunocompetence to the dental pulp [56]. They are expressed by both immune and non-immune cells in the pulp including neurons, fibroblasts, endothelial cells, epithelial cells and others, which recognize viral and microbial structures as well as self molecules (such as single stranded RNAs) that may accumulate in non-physiologic amounts or sites during inflammation [96–100].

Under normal conditions, very few immune cells are present in the dental pulp [101]. In the presence of infection (i.e. caries), immune cells are recruited to the pulp even in the absence of direct bacterial contact on the pulp tissue itself. The permeability of dentin to soluble bacterial products allows pulpal response to occur prior to carious pulpal exposure. These soluble bacterial products, along with components of the complement system and products of the lipoxygenase pathway of arachidonic acid metabolism are chemotactic for leukocytes [102].

The exponential increase in the number of infiltrating leukocytes brings with it a corresponding increase in lysosomal enzymes that cause tissue damage. Proteases like elastase and MMPs (Tables 1 and 2) cleave elastin and proteoglycans that destroy the pulp tissue resulting in irreversible damage [33, 58, 63]. Furthermore, the accompanying spike in inflammatory mediators like PGE2, cAMP, COX-2, CGRP, neurokinins and others stimulate vasodilation and microvascular permeability by binding into their respective receptors (i.e. EP2/3 receptor for PGE2) and induce cytoskeletal rearrangement or contraction of vascular smooth muscle [103].

Equally as important is the action of neuropeptides (e.g. substance P, calcitonin-gene related peptide) (Table 1). These neuropeptides typically reside in endings of afferent nerve close to blood vessels but also associated with macrophages and odontoblasts [104]. As a response to stimuli, afferent nerve sprouting has been demonstrated, and with it an increase in neuropeptide concentration [105], which can cause spontaneous pain, allodynia or hyperalgesia in teeth with pulpitis.

Simultaneous to the destructive effects of leukocytic infiltration is the capability of these cells to induce repair through the release of VEGF, TGF-B, GM-CSF and others (Tables 1 and 2) that induce alterations of the local extracellular matrix, promote induction of endothelial cells to migrate or proliferate, and inhibition of vascular growth with formation of differentiated capillaries [106]. The increased expression in inflamed pulp of toll-mediated human beta-defensins (hBD) [50] that play an important role in the innate host defense against bacterial invasion, contribute to promotion of adaptive immune responses, and show chemotactic activities further underscore the dynamic range of response of the dental pulp during inflammation. In addition, it can also be appreciated that during pulpal inflammation, the anti-inflammatory effects of various mediators such as tissue inhibitors of matrix proteinases (TIMPs), siRNA [94, 107] and others also come into play.

As a direct result of the release of inflammatory biomarkers, pulpal responses include classical signs of inflammation specifically a vascular response, along with changes in mediator profiles and cellular constituents. The transition from reversible to irreversible pulpitis has been broadly characterized by a migration of dendritic cells towards odontoblasts and accumulation of immune cells [108]. However, a more detailed analysis such in the majority of studies included in this paper evaluating biomarkers of pulpal inflammation demonstrates (statistical) significant differences between a clinically diagnosed healthy or irreversibly inflamed pulp at the molecular level.

**Fig 4:**
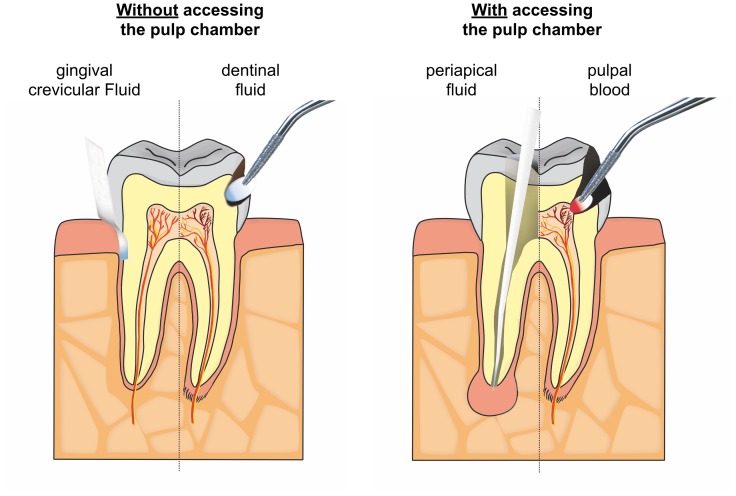
Actual and potential sampling sites to assay pulpal mediators.

Moreover, the analytes were obtained via different approaches both from the pulp directly as well as indirectly from tissue fluid. Fig 4 illustrates the potential sampling sites for molecular pulpal diagnostics [34]. While having the benefit to show a direct picture of intrapulpal conditions using pulpal blood [26] or whole pulp tissue requires access to the pulp space and is therefore not applicable as a chairside screening tool. Conversely, indirect methods such as dentin fluid collection or assessment of mediators in GCF can be performed clinical in a less invasive way. Dentin fluid is the extracellular fluid that is contained within dentinal tubules [109]; its composition includes inflammatory mediators and vasoactive compounds associated with inflammation. While initial evidence suggested that these mediators can be assayed [33] problems exist with protein yield [27] and the need to remove the existing restoration or in other cases to prepare an initial cavity deep in dentin.

GCF was used to sample mediators in one study included here (Table 2) [1]. This fluid is an exudate that from the gingival crevice and it contains several host factors including antibodies, bacterial antigens, proteins, and cytokines [110, 111]. GCF analyses may be promising due to the ease of application. Moreover, it may be possible to assess the dynamics of apical periodontitis using GCF [34]. However, the major drawback in the evaluation of host mediators in GCF is that tissue inflammation, independent of its cause, is a non-specific process of innate immunity [112] and this makes it difficult to distinguish on a molecular level between a marginal and apical periodontal inflammation. When sampling from the GCF for pulpal diagnostics, this potential drawback could be overcome by (i) creating healthy periodontal conditions, (ii) averaging out several sites on one or multiple teeth, (iii) combining clinical and radiographic observations, or (iv) defining a specific pattern of metabolites relevant to the pulp and not the periodontium, or possibly by other as yet unidentified means. Furthermore, the detection of mediators of pulpitis in GCF is impacted by the need for these compounds to reach the periodontal ligament and ultimately the gingival crevice in sufficient concentrations. Indeed, the notion that mediators will diffuse from the pulp via dentinal tubules or accessory canals to the periodontal ligament has been called into question [87]. Periapical fluid samples, while requiring a direct access to the apical site, are of interest to determine the level of systemic inflammation [28].

Discovering an improved method to determine the present inflammatory condition of the pulp could be of great value: on the one hand, pulp necrosis is one of the most frequent complications after coronal restoration of assumed non-inflamed (vital) teeth, on the other performing a full pulpectomy on teeth that could have been kept vital (at least in part) suggests that overtreatment may occur in many cases [113]. Endodontic diagnosis should therefore focus on either the extent of the microbial infection or the inflammatory reaction of the host tissue; however, current methods do neither [14, 23, 34].

Keeping a pulp vital offers distinct advantages compared to root canal treatment: the protective immune capacity of the pulp remains preserved and the remaining tooth structure gets not unnecessarily weakened by access cavity preparation and root canal enlargement. Unfortunately, the only available long-term outcome studies on direct pulp capping procedures (i.e. direct pulpal interventions), which attempt to maintain pulpal vitality, show unsatisfactory success rates as low as 20% after ten years [114]. The development of biocompatible materials facilitates a wound closure free of inflammation after pulpal capping procedures or partial pulpotomy [108]. However, the likelihood of a pulp to survive such procedures remains questionable using current schemes for assessment of pulpal inflammation.

One limitation of this systematic review is that merely 2 out of 57 studies [33, 45] were specifically designed to investigate potential biomarkers in the context of pulpal diagnostics. Most of the studies analyzed here merely target the presence of molecules and their function in pulpal inflammation. Nevertheless, based on the current state of knowledge this review provides an overview on molecules that are present and measurable during pulpal inflammation and therefore potentially can serve as a biomarker for pulpal inflammation. This may provide impulses for further research. This research needs to explore patient (age, gender, systemic condition) and infection related factors (varying composition of the microbiological infection). Clinical investigations should be conducted that are specifically designed to confirm the results collected from the research collected here. More specifically mediator profiles should be assessed in defined clinical scenarios. In addition, the assays methodology should be tested for their applicability with the possible substrates. The ultimate goal should then be to develop an inexpensive chairside test for non-invasive molecular pulp diagnostics. In fact, such a chair-side assay, based on the immunochromatographic detection of MMP-8 specific antibodies, is already commercial available to diagnose periodontal inflammation [115]. For endodontic procedures of the future, such as partial pulpotomies and pulp regeneration, a comparable test will be of significant value.

Indeed, various biomarkers that are produced by cellular components of the dental pulp can provide a snapshot of the biological mechanisms that propel this immunocompetent tissue towards healing or necrosis. The imbalance between tissue destructive molecules like proteases and tissue inductive molecules like VEGF may serve as a diagnostic or prognostic tool for endodontic intervention. The challenge remains on developing a method to make these biomarkers readily measureable in a clinical setting.

## Conclusions

In the included studies, irreversible pulpitis was associated with different expression of various biomarkers compared to non-inflamed controls. These biomarkers were significantly expressed not only in pulp tissue, but also in gingival crevicular fluid that can be collected non-invasively and in dentin fluid that can be analyzed without extirpating the pulpal tissue. This may be used to accurately differentiate diseased from healthy pulp tissue. The main current challenges in the clinical application of biomarkers lie in the identification of biomarkers or biomarker subsets that reliably correlate with pulpal inflammation, the improvement of sample collection (substrate and protein yields), and their analysis (interference of the biomarkers with inflammation of other than pulpal origin). If these hurdles can be overcome, a more accurate pulpal diagnosis and more predictable vital pulp treatment regime may create better clinical outcomes.

## Supporting Information

S1 Table PRISMA Checklist. (DOCX)

S2 Table Example of search strategy employed for this literature review. (DOCX)

S3 Table Hits from the literature search obtained with the different databases. (DOCX)

S4 Table Studies excluded from the final analysis. (DOCX)

S5 Table Assessment of the study quality using the modified Newcastle Ottawa Scale. (DOCX)
