# Supplementary material for: Biological Markers for Pulpal Inflammation: A Systematic Review
Source: PLoS One. 2016 Nov 29;11(11):e0167289. doi: 10.1371/journal.pone.0167289 (PMC5127562; doi:10.1371/journal.pone.0167289)
Supplement: S2 Table — (DOCX) [file pone.0167289.s002.docx]

**S2 Table** Example of search strategy (here in Medline database) employed for
 this literature review

| Number | Search history | Results |
| --- | --- | --- |
| 1 | (Exp Pulpitis/ OR ((acute or irreversible or painful) adj3 pulpitis).tw. OR (pulp* adj3 inflam*).tw.) | 2884 |
| 2 | dentinal fluid/ or gingival crevicular fluid/ OR  exp metalloendopeptidases/ or exp matrix metalloproteinases/ OR exp Biological Markers/an, ch, du, me OR exp inflammation mediators/ or exp "intercellular signaling peptides and proteins"/ OR "Osteoprotegerin"/ OR exp receptors, cytokine/ or osteoprotegerin/ OR *"Intracellular Signaling Peptides and Proteins"/ OR *"Lymphocytes"/ OR exp "Intracellular Signaling Peptides and Proteins"/ OR exp Antimicrobial Cationic Peptides/ | 1724182 |
| 3 | ((pulp* or dentin*) adj3 (tissue or fluid or liquor or supernatant)).tw. | 2423 |
| 4 | (gingival adj3 fluid).tw. | 2275 |
| 5 | biomarker.tw | 50450 |
| 6 | ((biological or diagnostic or inflammat*) adj3 (marker or mediator)).tw. | 17323 |
| 7 | (cytokin* or interferon* or tgf* or tnf* or interleukin* or metalloproteinase* or elastase* or lactoferrin or osteoprotegerin).tw. | 580865 |
| 8 | (antimicrobial adj3 peptides).tw. | 6692 |
| 9 | OR/2-7 | 1909267 |
| 10 | 1 AND 9 | 563 |
| 11 | 10 NOT (exp Cells, Cultured/ OR (animals not humans).sh.) | 347 |
