# Supplementary material for: Biological Markers for Pulpal Inflammation: A Systematic Review
Source: PLoS One. 2016 Nov 29;11(11):e0167289. doi: 10.1371/journal.pone.0167289 (PMC5127562; doi:10.1371/journal.pone.0167289)
Supplement: S3 Table — (DOCX) [file pone.0167289.s003.docx]

**S3 Table** Hits from the literature search obtained with the different databases

| Database | Inception of database | Hits | After duplicate removal |
| --- | --- | --- | --- |
| Medline | 1984 | 347 | 343 |
| Embase | 1974 | 334 | 88 |
| Cochrane | 1995 | 17 | 9 |
| ISI Web of Science | 1899 | 430 | 237 |
| Biosis | 1980 | 181 | 64 |
| Scopus  Pooled hits | 1996 | 420  1729 | 106  847 |
