# Supplementary material for: Biological Markers for Pulpal Inflammation: A Systematic Review
Source: PLoS One. 2016 Nov 29;11(11):e0167289. doi: 10.1371/journal.pone.0167289 (PMC5127562; doi:10.1371/journal.pone.0167289)
Supplement: S4 Table — (DOCX) [file pone.0167289.s004.docx]

**S4 Table** Studies excluded from the final analysis

| *Studies that did not use human teeth for analysis:* |
| --- |
| Mutoh et al. 2007 [1] |
|  |
| *Studies on cell cultures only:* |
| Hahn and Falkler 1992 [2], Casasco et al. 1994 [3], Barkhordar et al. 2002 [4], Lu et al. 2002 [5], Huang et al. 2006 [6], Huang et al. 2008 [7], Killough et al. 2009 [8], Lin et al. 2009 [9], Alongi et al. 2010 [10], Sattari et al. 2010 [11], Wang et al. 2010 [12], Hui et al. 2014 [13] |
|  |
| *No potential biomarker was investigated or the study was off topic:* |
| Maita and Horiuchi 1990 [14], Knutsson et al. 1994 [15], Tasman et al. 1999 [16], Wahlgren et al. 2002 [17], Rowland et al. 2007 [18], Avellan et al. 2008 [19], Rodriguez and Calero 2008 [20], Sattari et al. 2009 [21], Beneng et al. 2010 [22], Abd-Elmeguid et al. 2012 [23], Pereira et al. 2012 [24], Ruparel et al. 2013 [25] |
|  |
| No clear distinction between reversible, irreversible or necrotic pulp: |
| Pulver et al. 1977 [26], Speer et al. 1977 [27], Messelt et al. 1978 [28], Viti et al. 1985 [29], Gregoire and Terrie 1990 [30], Davis et al. 1991 [31], Grossi et al. 1991 [32], McClanahan et al. 1991 [33], Proctor et al. 1991 [34], Rauschenberger et al. 1991 [35], Skaljac-Staudt et al. 1991 [36], Rauschenberger, McClanahan et al. 1994 [37], Goodis and Saeki 1997 [38], Sawa et al. 1998 [39], Barkhordar et al. 1999 [40], Guo et al. 2000 [41], Hahn et al. 2000 [42], Ohnishi et al. 2000 [43], Rodd and Boissonade 2000 [44], Kuntz et al. 2001 [45], Nup et al. 2001 [46], Jukic et al. 2002 [47], Shin et al. 2002 [48], Pimenta et al. 2003 [49], McLachlan et al. 2004 [50], Paakkonen et al. 2005 [51], Dourou et al. 2006 [52], El Karim et al. 2006, [53] Rodd and Boissonade 2006 [54], El Karim et al. 2008 [55], Wang et al. 2008 [56], Warren et al. 2008 [57], Zhang et al. 2008 [58], Vavilova et al. 2009 [59], Cardoso et al. 2010 [60], Korkmaz et al. 2011 [61], Lee et al. 2011 [62], Zhao et al. 2011 [63], Evrosimovska et al. 2012 [64], Petrini et al. 2012 [65], Martin-Gonzalez et al. 2013 [66] |
|  |
|  |
| *Studies rather on histologic features or presence of cells, bacteria or viruses than on quantification of a biomarker:* |
| Pekovic and Fillery 1984 [67], Jacoby et al. 1991 [68], Casasco et al. 1992 [69], Izumi et al. 1995 [70], Wu and Wen 1995 [71], Kobayashi et al. 1996 [72], Marchetti 1996 [73], Zerosi et al. 1999 [74], Mousavi et al. 2006 [75], Pisterna and Siragusa 2007 [76], Manolea et al. 2008 [77], Rosaline et al. 2009 [78], Bruno et al. 2010 [79], Marigo et al. 2010 [80] |
|  |
|  |
| *Review articles, editorials, comments, abstract only or case reports:* |
| Ahlberg 1978 [81], Zelante et al. 1980 [82], D'Souza and Lachman 1989 [83], Jackson et al. 1992 [84], Rauschenberger and Cootauco 1993 [85], Goodis et al. 1994 [86], Matsuo et al. 1994 [87], Rauschenberger, Cootauco et al. 1994 [88], Taniishii et al. 1994 [89], Maclean et al. 1995 [90], Potente et al. 1998 [91], Huang et al. 2000 [92], Lopez-Costa et al. 2000 [93], Chidchuangchai et al. 2003 [94], Higuera 2012 [95], Martin Gonzalez et al. 2012 [96], Martin Jimenez et al. 2012 [97], Sanchez Dominguez 2012 [98], Segura Egea et al. 2012 [99] |

**References**

1. Mutoh N, Tani-Ishii N, Tsukinoki K, Chieda K, Watanabe K. Expression of toll-like receptor 2 and 4 in dental pulp. J Endod. 2007;33: 1183-1186. doi: 10.1016/j.joen.2007.05.018

2. Hahn CL, Falkler WA. Antibodies in normal and diseased pulps reactive with microorganisms isolated from deep caries. J Endod. 1992;18: 28-31. doi: 10.1016/s0099-2399(06)81139-8

3. Casasco A, Maserati E, Giordano M, Casasco M, Ciuffreda M, Sander S, et al. Stimulation of DNA synthesis by endothelin-1 in primary cultures of human dental pulp. Arch Oral Biol. 1994;39: 245-249. PMID: 8018054

4. Barkhordar RA, Ghani QP, Russell TR, Hussain MZ. Interleukin-1 beta activity and collagen synthesis in human dental pulp fibroblasts. J Endod. 2002;28: 157-159. doi: 10.1097/00004770-200203000-00003

5. Lu HX, Xiao MZ, Niu ZY, Guo XM, Zhao SL, Wang HG, et al. Effect of IL-1ra on human dental pulp cells and pulpal inflammation. Int Endod J. 2002;35: 807-811. doi: 10.1046/j.1365-2591.2002.00542.x

6. Huang FM, Tsai CH, Chen YJ, Chou MY, Chang YC. Examination of the signal transduction pathways leading to upregulation of tissue type plasminogen activator by interleukin-1 alpha in human pulp cells. J Endod. 2006;32: 30-33. doi: 10.1016/j.joen.2005.10.004

7. Huang GT, Lee HW, Lee HS, Lee GH, Huh SY, Choi GW, et al. Localization of substance P-induced upregulated interleukin-8 expression in human dental pulp explants. Int Endod J. 2008;41: 100-107. PMID: 18005045

8. Killough SA, Lundy HT, Irwin CR. Substance P Expression by Human Dental Pulp Fibroblasts: A Potential Role in Neurogenic Inflammation. J Endod. 2009;35: 73-77. doi: 10.1016/j.joen.2008.10.010

9. Lin ZM, Song Z, Qin W, Li J, Li WJ, Zhu HY, et al. Expression of Nucleotide-Binding Oligomerization Domain 2 in Normal Human Dental Pulp Cells and Dental Pulp Tissues. J Endod. 2009;35: 838-842. doi: 10.1016/j.joen.2009.03.047

10. Alongi DJ, Yamaza T, Song Y, Fouad AF, Romberg EE, Shi S, et al. Stem/progenitor cells from inflamed human dental pulp retain tissue regeneration potential. Regen Med. 2010;5: 617-631. doi: 10.2217/rme.10.30 PMID: 20465527

11. Sattari M, Mozayeni MA, Matloob A, Mozayeni M, Javaheri HH. Substance P and CGRP expression in dental pulps with irreversible pulpitis. Aust Endod J. 2010;36: 59-63. doi: 10.1111/j.1747-4477.2009.00186.x PMID: 20666750

12. Wang ZY, Pan J, Wright JT, Bencharit S, Zhang SP, Everett ET, et al. Putative Stem Cells in Human Dental Pulp with Irreversible Pulpitis: An Exploratory Study. J Endod. 2010;36: 820-825. doi: 10.1016/j.joen.2010.02.003

13. Hui T, A P, Zhao Y, Wang C, Gao B, Zhang P, et al. EZH2, a potential regulator of dental pulp inflammation and regeneration. J Endod. 2014;40: 1132-1138. doi: 10.1016/j.joen.2014.01.031 PMID: 25069920

14. Maita E, Horiuchi H. Polyamine analysis of infected root canal contents related to clinical symptoms. Endod Dent Traumatol. 1990;6: 213-217. PMID: 2133312

15. Knutsson G, Jontell M, Bergenholtz G. Determination of plasma-proteins in dental fluid from cavities prepared in healthy-young human teeth. Arch Oral Biol. 1994;39: 185-190. doi: 10.1016/0003-9969(94)90043-4

16. Tasman F, Dagdeviren A, Kendir B, Ozcelik B, Atac A, Er N. Endothelial cell adhesion molecules in human dental pulp: a comparative immunohistochemical study on chronic periodontitis. J Endod. 1999;25: 664-667. PMID: 10687524

17. Wahlgren J, Salo T, Teronen O, Luoto H, Sorsa T, Tjaderhane L. Matrix metalloproteinase-8 (MMP-8) in pulpal and periapical inflammation and periapical root-canal exudates. Int Endod J. 2002;35: 897-904. PMID: 12453017

18. Rowland KC, Kanive CB, Wells JE, Hatton JF. TRPM2 immunoreactivity is increased in fibroblasts, but not nerves, of symptomatic human dental pulp. J Endod. 2007;33: 245-248. PMID: 17320705

19. Avellan NL, Sorsa T, Tervahartiala T, Forster C, Kemppainen P. Experimental tooth pain elevates substance P and matrix metalloproteinase-8 levels in human gingival crevice fluid. Acta Odontol Scand. 2008;66: 18-22. doi: 10.1080/00016350701810658

20. Rodriguez P, Calero JA. Pulp microbiology of complete teeth with idiopathic apical lesions. Colomb Med. 2008;39: 5-10.

21. Sattari M, Haghighi AK, Tamijani HD. The relationship of pulp polyp with the presence and concentration of immunoglobulin E, histamine, interleukin-4 and interleukin-12. Aust Endod J. 2009;35: 164-168. doi: 10.1111/j.1747-4477.2009.00160.x PMID: 19961456

22. Beneng K, Renton T, Yilmaz Z, Yiangou Y, Anand P. Sodium channel Na v 1.7 immunoreactivity in painful human dental pulp and burning mouth syndrome. BMC Neurosci. 2010;11: 71. doi: 10.1186/1471-2202-11-71 PMID: 20529324

23. Abd-Elmeguid A, Yu DC, Kline LW, Moqbel R, Vliagoftis H. Dentin matrix protein-1 activates dental pulp fibroblasts. J Endod. 2012;38: 75-80. doi: 10.1016/j.joen.2011.10.005 PMID: 22152625

24. Pereira LO, Rubini MR, Silva JR, Oliveira DM, Silva ICR, Pocas-Fonseca MJ, et al. Comparison of stem cell properties of cells isolated from normal and inflamed dental pulps. Int Endod J. 2012;45: 1080-1090. doi: 10.1111/j.1365-2591.2012.02068.x

25. Ruparel S, Hargreaves KM, Eskander M, Rowan S, De Almeida JFA, Roman L, et al. Oxidized linoleic acid metabolite-cytochrome P450 system (OLAM-CYP) is active in biopsy samples from patients with inflammatory dental pain. Pain. 2013;154: 2363-2371.

26. Pulver WH, Taubman MA, Smith DJ. Immune components in normal and inflamed human dental pulp. Arch Oral Biol. 1977;22: 103-111.

27. Speer MI, Madonia JV, Heuer MA. Quantitative evaluation of the immunocompetence of the dental pulp. J Endod. 1977;3: 418-423.

28. Messelt EB, Skogedal O, Eriksen HM. Lactate dehydrogenase (LDH) isoenzyme pattern in normal and inflamed human dental pulp. Acta Odontol Scand. 1978;36: 345-348.

29. Viti M, Barucchi AM, Leprini A, Mazza A, Gaviano E. Activity of a nonspecific esterase (ANAE) on inflamed human pulp tissue. Parodontol Stomatol (Nuova). 1985;24: 25-30. PMID: 3939009

30. Gregoire G, Terrie B. Identification of lymphocyte antigens in human dental pulps. J Oral Pathol Med. 1990;19: 246-250. PMID: 1698220

31. Davis WL, Jacoby BH, Craig KR, Wagner G, Harrison JW. Copper-zinc superoxide dismutase activity in normal and inflamed human dental pulp tissue. J Endod. 1991;17: 316-318. PMID: 1779216

32. Grossi GB, Borrello S, Giuliani M, Galeotti T, Miani C. Copper-zinc superoxide dismutase in human and animal dental pulp. J Dent. 1991;19: 319-321. PMID: 1666897

33. McClanahan SB, Turner DW, Kaminski EJ, Osetek EM, Heuer MA. Natural modifiers of the inflammatory process in the human dental pulp. J Endod. 1991;17: 589-593. PMID: 1726472

34. Proctor ME, Turner DW, Kaminski EJ, Osetek EM, Heuer MA. Determination and relationship of c-reactive protein in human dental pulps and in serum. J Endod. 1991;17: 265-270. doi: 10.1016/s0099-2399(06)81864-9

35. Rauschenberger CR, Turner DW, Kaminski EJ, Osetek EM. Human polymorphonuclear granule components: relative levels detected by a modified enzyme-linked immunosorbent assay in normal and inflamed dental pulps. J Endod. 1991;17: 531-536. PMID: 1812201

36. Skaljac-Staudt G, Ciglar I, Sutalo J, Cvoriscec D. Quantitative evaluation of the immunoglobulin G, A and M in the human dental pulp. Acta Stomatol Croat. 1991;25: 33-38. PMID: 1819926

37. Rauschenberger CR, McClanahan SB, Pederson ED, Turner DW, Kaminski EJ. Comparison of human polymorphonuclear neutrophil elastase, polymorphonuclear neutrophil cathepsin-G, and alpha 2-macroglobulin levels in healthy and inflamed dental pulps. J Endod. 1994;20: 546-550. PMID: 7543925

38. Goodis H, Saeki K. Identification of bradykinin, substance P, and neurokinin A in human dental pulp. J Endod. 1997;23: 201-203.

39. Sawa Y, Yoshida S, Shibata KI, Suzuki M, Mukaida A. Vascular endothelium of human dental pulp expresses diverse adhesion molecules for leukocyte emigration. Tissue Cell. 1998;30: 281-291. PMID: 9661300

40. Barkhordar RA, Hayashi C, Hussain MZ. Detection of interleukin-6 in human dental pulp and periapical lesions. Endod Dent Traumatol. 1999;15: 26-27. PMID: 10219150

41. Guo X, Niu Z, Xiao M, Yue L, Lu H. Detection of interleukin-8 in exudates from normal and inflamed human dental pulp tissues. Chin J Dent Res. 2000;3: 63-66. PMID: 11314344

42. Hahn CL, Best AM, Tew JG. Cytokine induction by Streptococcus mutans and pulpal pathogenesis. Infect Immun. 2000;68: 6785-6789. PMID: 11083796

43. Ohnishi T, Suwa M, Oyama T, Arakaki N, Torii M, Daikuhara Y. Prostaglandin E-2 predominantly induces production of hepatocyte growth factor/scatter factor in human dental pulp in acute inflammation. J Dent Res. 2000;79: 748-755.

44. Rodd HD, Boissonade FM. Substance P expression in human tooth pulp in relation to caries and pain experience. Eur J Oral Sci. 2000;108: 467-474. PMID: 11153921

45. Kuntz KA, Brown CE, Jr., Legan JJ, Kafrawy AH. An immunohistochemical study of osteoprotegerin in the human dental pulp. J Endod. 2001;27: 666-669. PMID: 11716077

46. Nup C, Rosenberg P, Linke H, Tordik P. Quantitation of catecholamines in inflamed human dental pulp by high-performance liquid chromatography. J Endod. 2001;27: 73-75. PMID: 11491641

47. Jukic S, Talan-Hranilovic J, Bukovic D, Miletic I, Neziri E. Nicotinamide adenine dinucleotide phosphate-diaphorase (NADPH-d) histochemistry detecting NOS in healthy and chronically inflamed pulp. Coll Antropol. 2002;26: 681-688. PMID: 12528299

48. Shin SJ, Lee JI, Baek SH, Lim SS. Tissue levels of matrix metalloproteinases in pulps and periapical lesions. J Endod. 2002;28: 313-315. PMID: 12043871

49. Pimenta FJ, Sa AR, Gomez RS. Lymphangiogenesis in human dental pulp. Int Endod J. 2003;36: 853-856. PMID: 14641424

50. McLachlan JL, Sloan AJ, Smith AJ, Landini G, Cooper PR. S100 and cytokine expression in caries. Infect Immun. 2004;72: 4102-4108. PMID: 15213155

51. Paakkonen V, Ohlmeier S, Bergmann U, Larmas M, Salo T, Tjaderhane L. Analysis of gene and protein expression in healthy and carious tooth pulp with cDNA microarray and two-dimensional gel electrophoresis. Eur J Oral Sci. 2005;113: 369-379. PMID: 16202023

52. Dourou V, Lyroudia K, Karayannopoulou G, Papadimitriou C, Molyvdas I. Comparative evaluation of neural tissue antigens--neurofilament protein (NF), peripherin (PRP), S100B protein (S100B), neuron-specific enolase (NSE) and chromogranin-A (CgA)--in both normal and inflamed human mature dental pulp. Acta Histochem. 2006;108: 343-350. PMID: 16919707

53. El Karim IA, Lamey PJ, Linden GJ, Awawdeh LA, Lundy FT. Caries-induced changes in the expression of pulpal neuropeptide Y. Eur J Oral Sci. 2006;114: 133-137. PMID: 16630305

54. Rodd HD, Boissonade FM. Immunocytochemical investigation of immune cells within human primary and permanent tooth pulp. Int J Paediatr Dent. 2006;16: 2-9. PMID: 16364087

55. El Karim IA, Lamey PJ, Linden GJ, Lundy FT. Neuropeptide Y Y1 receptor in human dental pulp cells of noncarious and carious teeth. Int Endod J. 2008;41: 850-855. doi: 10.1111/j.1365-2591.2008.01436.x PMID: 18699789

56. Wang X, Zhang Q, Chen Z, Zhang L. Immunohistochemical localization of LIM mineralization protein 1 in pulp-dentin complex of human teeth with normal and pathologic conditions. J Endod. 2008;34: 143-147. doi: 10.1016/j.joen.2007.10.015 PMID: 18215669

57. Warren CA, Mok L, Gordon S, Fouad AF, Gold MS. Quantification of neural protein in extirpated tooth pulp. J Endod. 2008;34: 7-10. PMID: 18155483

58. Zhang ZM, Gao X, Wang CK, Zhang YY. Expression of monocyte chemoattractant protein-1 in pulp tissue in patients with pulpitis and its significance. Journal of Jilin University Medicine Edition. 2008;34: 1035-1037.

59. Vavilova T, Ostrovskaya I, Axenova L, Buneeva O, Medvedev A. Monoamine oxidase and semicarbazide sensitive amine oxidase activities in normal and inflamed human dental pulp. Med Sci Monit. 2009;15: BR289-292. PMID: 19789505

60. Cardoso FP, Viana MB, Sobrinho AP, Diniz MG, Brito JA, Gomes CC, et al. Methylation pattern of the IFN-gamma gene in human dental pulp. J Endod. 2010;36: 642-646. doi: 10.1016/j.joen.2009.12.017 PMID: 20307737

61. Korkmaz Y, Lang H, Beikler T, Cho B, Behrends S, Bloch W, et al. Irreversible inflammation is associated with decreased levels of the alpha1-, beta1-, and alpha2-subunits of sGC in human odontoblasts. J Dent Res. 2011;90: 517-522. doi: 10.1177/0022034510390808 PMID: 21212316

62. Lee YY, Chan CH, Hung SL, Chen YC, Lee YH, Yang SF. Up-regulation of Nucleotide-binding Oligomerization Domain 1 in Inflamed Human Dental Pulp. J Endod. 2011;37: 1370-1375. doi: 10.1016/j.joen.2011.06.008

63. Zhao DF, Gong QM, Ling JQ, Zhang XF. Macrophage migration-inhibitory factors expression and its effects on proliferation in human dental pulps. Chung-Hua Kou Chiang i Hsueh Tsa Chih Chinese Journal of Stomatology. 2011;46: 484-488. PMID: 22169746

64. Evrosimovska B, Dimova C, Kovacevska I, Panov S. Concentration of collagenases (MMP-1, -8, -13) in patients with chronically inflamed dental pulp tissue. Makedonska Akademija na Naukite i Umetnostite Oddelenie Za Bioloshki i Meditsinski Nauki Prilozi. 2012;33: 191-204. PMID: 23425881

65. Petrini M, Ferrante M, Ciavarelli L, Brunetti L, Vacca M, Spoto G. Prostaglandin E2 to diagnose between reversible and irreversible pulpitis. Int J Immunopathol Pharmacol. 2012;25: 157-163. PMID: 22507328

66. Martin-Gonzalez J, Sanchez-Jimenez F, Perez-Perez A, Carmona-Fernandez A, Sanchez-Margalet V, Segura-Egea JJ. Leptin expression in healthy and inflamed human dental pulp. Int Endod J. 2013;46: 442-448. doi: 10.1111/iej.12009

67. Pekovic DD, Fillery ED. Identification of bacteria in immunopathologic mechanisms of human dental pulp. Oral surgery, oral medicine, and oral pathology. 1984;57: 652-661.

68. Jacoby BH, Davis WL, Craig KR, Wagner G, Farmer GR, Harrison JW. An ultrastructural and immunohistochemical study of human dental-pulp - identification of weibel-palade bodies and von willebrand factor in pulp endothelial cells. J Endod. 1991;17: 150-155.

69. Casasco A, Casasco M, Ciuffreda M, Springall DR, Calligaro A, Bianchi S, et al. Immunohistochemical evidence for the occurrence of endothelin in the vascular endothelium of normal and inflamed human dental pulp. J Dent Res. 1992;71: 475-477. PMID: 1573079

70. Izumi T, Kobayashi I, Okamura K, Sakai H. Immunohistochemical study on the immunocompetent cells of the pulp in human non-carious and carious teeth. Arch Oral Biol. 1995;40: 609-614. PMID: 7575232

71. Wu H, Wen L. Immunohistochemical study of T and B cells in dental pulp. Zhonghua Kou Qiang Yi Xue Za Zhi. 1995;30: 346-348.

72. Kobayashi I, Izumi T, Okamura K, Matsuo K, Ishibashi Y, Sakai H. Biological behavior of human dental pulp cells in response to carious stimuli analyzed by PCNA immunostaining and AgNOR staining. Caries Res. 1996;30: 225-230. PMID: 8860034

73. Marchetti C. Weibel-Palade bodies and lymphatic endothelium: observations in the lymphatic vessels of normal and inflamed human dental pulps. Vasa. 1996;25: 337-340. PMID: 8956550

74. Zerosi C, Giardino L, Lafornara D, Scarola V. Presence of Russel bodies in chronic dental pulp inflammation. Minerva Stomatol. 1999;48: 307-310.

75. Mousavi SB, Talebi A, Kianoosh S. Immunohistochemical assessment of natural killer cells in normal and inflamed dental pulps. J Res Med Sci. 2006;11: 119-121.

76. Pisterna GV, Siragusa M. CD44 Presence in inflamed pulp tissue. J Endod. 2007;33: 1203-1207. PMID: 17889690

77. Manolea H, Mogoanta L, Margaritescu C, Deva V, Surlin P, Caraivan O. Immunohistochemical aspects of the evaluation of the inflammatory answer of the dental pulp. Rom J Morphol Embryol. 2008;50: 207-212.

78. Rosaline H, Satish ES, Kandaswamy D. Detection of presence or absence of herpes simplex virus, Epstein Barr virus and human cytomegalovirus in infected pulp using a polymerase chain reaction. Aust Endod J 2009;35: 9-12. PMID: 19452674

79. Bruno KF, Silva JA, Silva TA, Batista AC, Alencar AH, Estrela C. Characterization of inflammatory cell infiltrate in human dental pulpitis. Int Endod J. 2010;43: 1013-1021. doi: 10.1111/j.1365-2591.2010.01757.x PMID: 20726912

80. Marigo L, Migliaccio S, Monego G, La Torre G, Somma F, Ranelletti FO. Expression of parathyroid hormone-related protein in human inflamed dental pulp. Eur Rev Med Pharmacol Sci. 2010;14: 471-475. PMID: 20556927

81. Ahlberg KF. Functional studies on experimentally induced inflammations of the dental pulp. Tandlakartidningen 1978;70: 837-838. PMID: 284603

82. Zelante F, Simões W, Monson CA. Participation of immunologic responses in evaluation of pulp and periapical inflammation. Rev Assoc Paul Cir Dent. 1980;34: 420-425. PMID: 6450437

83. D'Souza R, Lachman LB. Levels of Interleukins 1 and 2 in Inflamed Human Pulps. J Dent Res. 1989;68: 201.

84. Jackson D, Garry M, Engelstad M, Geier H, Hargreaves K. Evaluation of iCGRP secretion from dental pulp in response to inflammatory mediators. Society for Neuroscience Abstracts. 1992;18: 689.

85. Rauschenberger RA, Cootauco C. The immunocytochemical distribution of IL-2R in healthy and inflamed dental pulp. J Dent Res. 1993;72: 212.

86. Goodis HE, White JM, Gerzina TM, Hume WR. Identification of pulpal inflammatory mediators using HPLC. J Dent Res. 1994;73: 214-214.

87. Matsuo T, Nakanishi T, Ebisu S. Immunoglobulins and inflammatory factors in human pulpal blood J Dent Res. 1994;73: 214-214.

88. Rauschenberger C, Cootauco C, Bailey J. Interleukin-2 receptor (SIL-2R) in healty and inflamed dental pulps. J Dent Res. 1994;73: 120-120.

89. Taniishii N, Wang C, Stashenko P. Bone resorptive cytokine production in inflamed pulp and periapical lesions. J Dent Res. 1994;73: 119-119.

90. Maclean DC, Bowen L, Langkamp H, Agarwal S, Piesco NP. Regulation of cytokines in pulp cells during inflammation. J Dent Res. 1995;74: 181-181.

91. Potente AP, Chugal N, Kim JW, Zhang X, Huang GTJ. Detection of interleukin-8 in normal and inflamed human dental pulps. J Dent Res. 1998;77: 799-799.

92. Huang GTJ, Chugal N, Potente AP, Zhang X. Constitutive expression of interleukin-8 and intercellular adhesion molecule-1 in normal and inflamed human dental pulps. J Dent Res. 2000;79: 176-176.

93. Lopez-Costa JJ, Colera-Martin L, Patel S, Maresca B, Saavedra JP. NADPH diaphorase reactivity in human dental pulp: role in pain and inflammation. Society for Neuroscience Abstracts. 2000;26: Abstract No.-731.734.

94. Chidchuangchai W, Akarasereenont P, Janyaprasert K, Vongsavan N, Matthews B. Prostaglandin E-2 levels, blood flow, and pain sensation in human teeth with inflamed pulps. J Dent Res. 2003;82: B249-B249.

95. Higuera J. Correlation between IL 8, 810 nm laser energy and clinical-radiographic diagnostic of periapical area tested with ELISA. Clinical study. Med Oral Patol Oral Cir Bucal. 2012;17: S141.

96. Martin Gonzalez J, Sanchez Dominguez B, Castellanos Cosano L, Torres Lagares D, Segura Egea JJ. Expression of leptin mRNA in normal human dental pulp. Med Oral Patol Oral Cir Bucal. 2012;17.

97. Martin Jimenez M, Martin Gonzalez J, Sanchez Dominguez B, Lopez Frias J, Segura Egea JJ. Expression of mRNA leptin receptor (Ob-R) in normal human dental pulp: Implications in pulp revascularization. Med Oral Patol Oral Cir Bucal. 2012;17.

98. Sanchez Dominguez B. Expression of leptin in normal human dental pulp. Med Oral Patol Oral Cir Bucal. 2012;17.

99. Segura Egea JJ, Martin Gonzalez J, Sanchez Dominguez B, Martin Jimenez M, Tarilonte Delgado ML. Presence of leptin receptor (Ob-R) in normal human dental pulp: Demons-tration by Western-Blo. Med Oral Patol Oral Cir Bucal. 2012;17.
