# Supplementary material for: Biological Markers for Pulpal Inflammation: A Systematic Review
Source: PLoS One. 2016 Nov 29;11(11):e0167289. doi: 10.1371/journal.pone.0167289 (PMC5127562; doi:10.1371/journal.pone.0167289)
Supplement: S5 Table — (DOCX) [file pone.0167289.s005.docx]

**S5 Table** Assessment of the study quality using the Modified Newcastle Ottawa Scale

|  | **Selection** | | **Comparability** | | | | **Outcome** | | **Total quality score** |
| --- | --- | --- | --- | --- | --- | --- | --- | --- | --- |
| Reference | Selection of the cohort | Condition of the cohort | Diagnostics of cases and controls | Pulpal inflammation confirmation histologically | Quality of the controls | Ratio of the group size (control:case) | Reported blinding to case/control status | Same tests performed for cases and controls |  |
| Bolanos and Seltzer 1981 [[82](#_ENREF_82)] | - | - | * | - | - | - | * | * | 3 |
| Cohen et al. 1985 [[47](#_ENREF_47)] | * | * | * | - | - | * | - | * | 5 |
| Nakanishi et al. 1995 [[44](#_ENREF_44)] | - | * | * | - | - | - | - | * | 3 |
| Cootauco et al. 1993 [[73](#_ENREF_73)] | - | - | * | * | - | * | - | * | 4 |
| Ge *et al.* 1996 [[69](#_ENREF_69)] | - | * | - | - | - | * | - | * | 3 |
| Rauschenberger et al. 1997 [[52](#_ENREF_52)] | - | * | * | * | - | * | - | * | 5 |
| Tulunoglu et al. 1998 [[70](#_ENREF_70)] | - | * | * | - | - | * | - | * | 4 |
| Dong et al. 1999 [[83](#_ENREF_83)] | - | * | - | - | - | * | - | * | 3 |
| Huang et al. 1999 [[54](#_ENREF_54)] | - | * | * | * | - | * | - | * | 5 |
| Lepinski et al. 2000 [[41](#_ENREF_41)] | * | - | * | - | - | * | - | * | 4 |
| Khabbaz et al. 2001 [[84](#_ENREF_84)] | * | - | * | - | - | - | * | * | 4 |
| Nakanishi et al. 2001 [[85](#_ENREF_85)] | - | * | - | * | - | * | - | * | 4 |
| Spoto, Fioroni, Rubini, Tripodi, Di Stilio et al. 2001 [[74](#_ENREF_74)] | - | - | - | * | - | * | - | * | 3 |
| Spoto, Fioroni, Rubini, Tripodi, Perinetti et al. 2001 [[75](#_ENREF_75)] | - | - | - | - | - | * | - | * | 2 |
| Anderson et al. 2002 [[53](#_ENREF_53)] | - | - | * | - | - | * | * | * | 4 |
| Artese et al. 2002 [[91](#_ENREF_91)] | - | - | - | * | - | * | - | * | 3 |
| Awawdeh et al. 2002 [[87](#_ENREF_87)] | * | * | - | - | - | - | * | * | 4 |
| Gusman et al. 2002 [[63](#_ENREF_63)] | * | * | * | - | - | * | - | * | 5 |
| Pezelj-Ribaric et al. 2002 [[55](#_ENREF_55)] | - | - | * | - | - | * | - | * | 3 |
| Bowles et al. 2003 [[42](#_ENREF_42)] | - | - | * | - | - | * | - | * | 3 |
| Esposito, Varvara, Caputi et al. 2003 [[76](#_ENREF_76)] | * | * | * | - | - | * | * | * | 6 |
| Esposito, Varvara, Murmura et al. 2003 [[77](#_ENREF_77)] | * | * | * | - | - | * | - | * | 5 |
| Zehnder et al. 2003 [[48](#_ENREF_48)] | * | * | * | - | - | * | - | * | 5 |
| Caviedes-Bucheli et al. 2004 [[89](#_ENREF_89)] | - | - | * | - | - | * | - | * | 3 |
| Di Nardo Di Maio et al. 2004 [[79](#_ENREF_79)] | - | - | * | * | - | * | - | * | 4 |
| Piattelli et al. 2004 [[59](#_ENREF_59)] | - | - | - | * | - | * | * | * | 4 |
| Spoto, Ferrante et al. 2004 [[80](#_ENREF_80)] | - | - | - | - | - | * | - | * | 2 |
| Spoto, Menna et al. 2004 [[81](#_ENREF_81)] | - | - | - | - | - | * | - | * | 2 |
| Caviedes-Bucheli et al. 2005 [[90](#_ENREF_90)] | - | - | * | - | - | * | - | * | 3 |
| Huang et al. 2005 [[67](#_ENREF_67)] | - | * | * | * | - | * | - | * | 5 |
| Nakanishi et al. 2005 [[58](#_ENREF_58)] | - | * | - | * | - | * | - | * | 4 |
| Tsai et al. 2005 [[65](#_ENREF_65)] | - | * | * | * | - | * | - | * | 5 |
| Varvara et al. 2005 [[72](#_ENREF_72)] | * | * | * | - | - | * | - | * | 5 |
| Caviedes-Bucheli et al. 2006 [[88](#_ENREF_88)] | - | - | - | - | - | * | - | * | 2 |
| Evcil et al. 2006 [[46](#_ENREF_46)] | * | * | - | - | * | * | - | * | 5 |
| Adachi et al. 2007 [[60](#_ENREF_60)] | - | * | - | * | - | - | - | * | 3 |
| Bodor et al. 2007 [[71](#_ENREF_71)] | * | * | * | - | - | * | - | * | 5 |
| Caviedes-Bucheli et al. 2007 [[92](#_ENREF_92)] | * | - | - | - | - | * | - | * | 3 |
| Guven et al. 2007 [[86](#_ENREF_86)] | - | * | - | * | - | * | - | * | 4 |
| Huang et al. 2007 [[68](#_ENREF_68)] | - | * | - | * | - | - | - | * | 3 |
| Kokkas et al. 2007 [[43](#_ENREF_43)] | - | - | * | - | - | * | - | * | 3 |
| Caviedes-Bucheli, Moreno et al. 2008 [[93](#_ENREF_93)] | - | - | - | - | - | * | - | * | 2 |
| da Silva et al. 2008 [[78](#_ENREF_78)] | - | - | - | * | - | * | * | * | 4 |
| Jiang et al. 2008 [[61](#_ENREF_61)] | - | - | * | * | - | * | - | * | 4 |
| Karapanou et al. 2008 [[1](#_ENREF_1)] | * | * | * | - | * | * | - | * | 6 |
| Huang et al. 2009 [[62](#_ENREF_62)] | - | * | * | * | - | * | - | * | 5 |
| Keller et al. 2009 [[56](#_ENREF_56)] | - | - | * | - | - | * | - | * | 3 |
| Paris et al. 2009 [[50](#_ENREF_50)] | - | - | * | - | - | * | - | * | 3 |
| Silva et al. 2009 [[51](#_ENREF_51)] | - | - | * | * | - | * | * | * | 5 |
| Li et al. 2011 [[57](#_ENREF_57)] | * | * | - | * | - | * | - | * | 5 |
| Zehnder et al. 2011 [[33](#_ENREF_33)] | * | * | * | - | - | * | * | * | 6 |
| Suwanchai et al. 2012 [[66](#_ENREF_66)] | - | - | - | - | - | * | - | * | 2 |
| Zhong et al. 2012 [[94](#_ENREF_94)] | * | * | * | - | - | * | - | * | 5 |
| Abd-Elmeguid et al. 2013 [[49](#_ENREF_49)] | - | - | * | * | - | * | * | * | 5 |
| Accorsi-Mendonca et al. 2013 [[64](#_ENREF_64)] | - | * | * | - | - | * | - | * | 4 |
| Dong et al. 2013 [[95](#_ENREF_95)] | * | * | * | * | - | * | - | * | 6 |
| Elsalhy et al. 2013 [[45](#_ENREF_45)] | * | * | * | - | - | * | - | * | 5 |

**References**

1. Karapanou V, Kempuraj D, Theoharides TC. Interleukin-8 is increased in gingival crevicular fluid from patients with acute pulpitis. J Endod. 2008;34: 148-151. doi: 10.1016/j.joen.2007.10.022 PMID: 18215670

33. Zehnder M, Wegehaupt FJ, Attin T. A first study on the usefulness of matrix metalloproteinase 9 from dentinal fluid to indicate pulp inflammation. J Endod. 2011;37: 17-20. doi: 10.1016/j.joen.2010.10.003 PMID: 21146069

41. Lepinski AM, Hargreaves KM, Goodis HE, Bowles WR. Bradykinin levels in dental pulp by microdialysis. J Endod. 2000;26: 744-747. doi: 10.1097/00004770-200012000-00020 PMID: 11471646

42. Bowles WR, Withrow JC, Lepinski AM, Hargreaves KM. Tissue levels of immunoreactive substance P are increased in patients with irreversible pulpitis. J Endod. 2003;29: 265-267. doi: 10.1097/00004770-200304000-00009 PMID: 12701777

43. Kokkas AB, Goulas A, Varsamidis K, Mirtsou V, Tziafas D. Irreversible but not reversible pulpitis is associated with up-regulation of tumour necrosis factor-alpha gene expression in human pulp. Int Endod J. 2007;40: 198-203. doi: 10.1111/j.1365-2591.2007.01215.x PMID: 17305696

44. Nakanishi T, Matsuo T, Ebisu S. Quantitative analysis of immunoglobulins and inflammatory factors in human pulpal blood from exposed pulps. J Endod. 1995;21: 131-136. PMID: 7561655

45. Elsalhy M, Azizieh F, Raghupathy R. Cytokines as diagnostic markers of pulpal inflammation. Int Endod J. 2013;46: 573-580. doi: 10.1111/iej.12030 PMID: 23240887

46. Evcil MS, Keles A, Uzun I, Demircan B, Koseoglu M. Nitric oxide levels in serum of patients with symptomatic irreversible pulpitis. J Pain Palliat Care Pharmacother. 2006;20: 15-19. PMID: 16687351

47. Cohen JS, Reader A, Fertel R, Beck M, Meyers WJ. A radioimmunoassay determination of the concentrations of prostaglandins E2 and F2alpha in painful and asymptomatic human dental pulps. J Endod. 1985;11: 330-335. PMID: 3863874

48. Zehnder M, Delaleu N, Du Y, Bickel M. Cytokine gene expression--part of host defence in pulpitis. Cytokine. 2003;22: 84-88. PMID: 12849707

49. Abd-Elmeguid A, Abdeldayem M, Kline LW, Moqbel R, Vliagoftis H, Yu DC. Osteocalcin expression in pulp inflammation. J Endod. 2013;39: 865-872. doi: 10.1016/j.joen.2012.12.035 PMID: 23791253

50. Paris S, Wolgin M, Kielbassa AM, Pries A, Zakrzewicz A. Gene expression of human beta-defensins in healthy and inflamed human dental pulps. J Endod. 2009;35: 520-523. doi: 10.1016/j.joen.2008.12.015 PMID: 19345797

51. Silva AC, Faria MR, Fontes A, Campos MS, Cavalcanti BN. Interleukin-1 beta and interleukin-8 in healthy and inflamed dental pulps. J Appl Oral Sci. 2009;17: 527-532. PMID: 19936537

52. Rauschenberger CR, Bailey JC, Cootauco CJ. Detection of human IL-2 in normal and inflamed dental pulps. J Endod. 1997;23: 366-370. doi: 10.1016/S0099-2399(97)80184-7 PMID: 9545944

53. Anderson LM, Dumsha TC, McDonald NJ, Spitznagel JK, Jr. Evaluating IL-2 levels in human pulp tissue. J Endod. 2002;28: 651-655. doi: 10.1097/00004770-200209000-00006 PMID: 12236309

54. Huang GT, Potente AP, Kim JW, Chugal N, Zhang X. Increased interleukin-8 expression in inflamed human dental pulps. Oral Surg Oral Med Oral Pathol Oral Radiol Endod. 1999;88: 214-220. PMID: 10468466

55. Pezelj-Ribaric S, Anic I, Brekalo I, Miletic I, Hasan M, Simunovic-Soskic M. Detection of tumor necrosis factor alpha in normal and inflamed human dental pulps. Arch Med Res. 2002;33: 482-484. PMID: 12459320

56. Keller JF, Carrouel F, Staquet MJ, Kufer TA, Baudouin C, Msika P, et al. Expression of NOD2 is increased in inflamed human dental pulps and lipoteichoic acid-stimulated odontoblast-like cells. Innate immunity. 2009;17: 29-34. doi: 10.1177/1753425909348527 PMID: 19880660

57. Li NN, Zhang ZM, Wang CK, Wang JR, Meng XP. Expression of MIP-1(alpha) mRNA in inflammed pulp tissue and its significance. J Jilin Univ Med. 2011;37: 312-314.

58. Nakanishi T, Takahashi K, Hosokawa Y, Adachi T, Nakae H, Matsuo T. Expression of macrophage inflammatory protein 3alpha in human inflamed dental pulp tissue. J Endod. 2005;31: 84-87. PMID: 15671814

59. Piattelli A, Rubini C, Fioroni M, Tripodi D, Strocchi R. Transforming growth factor-beta 1 (TGF-beta 1) expression in normal healthy pulps and in those with irreversible pulpitis. Int Endod J. 2004;37: 114-119. PMID: 14871177

60. Adachi T, Nakanishi T, Yumoto H, Hirao K, Takahashi K, Mukai K, et al. Caries-related bacteria and cytokines induce CXCL10 in dental pulp. J Dent Res. 2007;86: 1217-1222. PMID: 18037659

61. Jiang HW, Ling JQ, Gong QM. The expression of stromal cell-derived factor 1 (SDF-1) in inflamed human dental pulp. J Endod. 2008;34: 1351-1354. doi: 10.1016/j.joen.2008.07.023 PMID: 18928845

62. Huang FM, Tsai CH, Yang SF, Chang YC. The upregulation of oncostatin M in inflamed human dental pulps. Int Endod J. 2009;42: 627-631. doi: 10.1111/j.1365-2591.2009.01567.x PMID: 19467046

63. Gusman H, Santana RB, Zehnder M. Matrix metalloproteinase levels and gelatinolytic activity in clinically healthy and inflamed human dental pulps. Eur J Oral Sci. 2002;110: 353-357. PMID: 12664465

64. Accorsi-Mendonca T, Silva EJ, Marcaccini AM, Gerlach RF, Duarte KM, Pardo AP, et al. Evaluation of gelatinases, tissue inhibitor of matrix metalloproteinase-2, and myeloperoxidase protein in healthy and inflamed human dental pulp tissue. J Endod. 2013;39: 879-882. doi: 10.1016/j.joen.2012.11.011 PMID: 23791255

65. Tsai CH, Chen YJ, Huang FM, Su YF, Chang YC. The upregulation of matrix metalloproteinase-9 in inflamed human dental pulps. J Endod. 2005;31: 860-862. PMID: 16306818

66. Suwanchai A, Theerapiboon U, Chattipakorn N, Chattipakorn SC. NaV 1.8, but not NaV 1.9, is upregulated in the inflamed dental pulp tissue of human primary teeth. Int Endod J. 2012;45: 372-378. doi: 10.1111/j.1365-2591.2011.01986.x PMID: 22085016

67. Huang FM, Tsai CH, Chen YJ, Liu CM, Chou MY, Chang YC. Upregulation of tissue-type plasminogen activator in inflamed human dental pulps. Int Endod J. 2005;38: 328-333. PMID: 15876297

68. Huang FM, Yang SF, Chen YJ, Tsai CH, Chang YC. Tissue type plasminogen activator level and caseinolytic activity in clinically healthy and inflamed human dental pulp. J Dent Sci. 2007;2: 152-156.

69. Ge J, Ji J, Wang T. Superoxide dismutase and malonyl dialdehyde in human pulp tissue. Chung Hua Kou Chiang Hsueh Tsa Chih. 1996;31: 201-203. PMID: 9592267

70. Tulunoglu O, Alacam A, Bastug M, Yavuzer S. Superoxide dismutase activity in healthy and inflamed pulp tissues of permanent teeth in children. J Clin Pediatr Dent. 1998;22: 341-345. PMID: 9796506

71. Bodor C, Matolcsy A, Bernath M. Elevated expression of Cu, Zn-SOD and Mn-SOD mRNA in inflamed dental pulp tissue. Int Endod J. 2007;40: 128-132. PMID: 17229118

72. Varvara G, Traini T, Esposito P, Caputi S, Perinetti G. Copper-zinc superoxide dismutase activity in healthy and inflamed human dental pulp. Int Endod J. 2005;38: 195-199. PMID: 15743423

73. Cootauco CJ, Rauschenberger CR, Nauman RK. Immunocytochemical distribution of human PMN elastase and cathepsin-G in dental pulp. J Dent Res. 1993;72: 1485-1490. PMID: 7693782

74. Spoto G, Fioroni M, Rubini C, Tripodi D, Di Stilio M, Piattelli A. Alkaline phosphatase activity in normal and inflamed dental pulps. J Endod. 2001;27: 180-182. doi: 10.1097/00004770-200103000-00010 PMID: 11487147

75. Spoto G, Fioroni M, Rubini C, Tripodi D, Perinetti G, Piattelli A. Aspartate aminotransferase activity in human healthy and inflamed dental pulps. J Endod. 2001;27: 394-395. PMID: 11487132

76. Esposito P, Varvara G, Caputi S, Perinetti G. Catalase activity in human healthy and inflamed dental pulps. Int Endod J. 2003;36: 599-603. PMID: 12950573

77. Esposito P, Varvara G, Murmura G, Terlizzi A, Caputi S. Ability of healthy and inflamed human dental pulp to reduce hydrogen peroxide. Eur J Oral Sci. 2003;111: 454-456. PMID: 12974692

78. da Silva LP, Issa JP, Del Bel EA. Action of nitric oxide on healthy and inflamed human dental pulp tissue. Micron. 2008;39: 797-801. doi: 10.1016/j.micron.2008.01.018 PMID: 18337111

79. Di Nardo Di Maio F, Lohinai Z, D'Arcangelo C, De Fazio PE, Speranza L, De Lutiis MA, et al. Nitric oxide synthase in healthy and inflamed human dental pulp. J Dent Res. 2004;83: 312-316. PMID: 15044505

80. Spoto G, Ferrante M, D'Intino M, Rega L, Dolci M, Trentini P, et al. Cyclic GMP phosphodiesterase activity role in normal and inflamed human dental pulp. Int J Immunopathol Pharmacol. 2004;17: 21-24. PMID: 16857102

81. Spoto G, Menna V, Serra E, Santoleri F, Perfetti G, Ciavarelli L, et al. Cyclic AMP phosphodiesterase activity in normal and inflamed human dental pulp. Int J Immunopathol Pharmacol. 2004;17: 11-15. PMID: 16857100

82. Bolanos OR, Seltzer S. Cyclic AMP and cyclic GMP quantitation in pulp and periapical lesions and their correlation with pain. J Endod. 1981;7: 268-271. doi: 10.1016/S0099-2399(81)80005-2 PMID: 6265580

83. Dong Y, Zhou J, Pan Y. Quantitive study of 6-keto-prostaglandin F1 alpha and thromboxane B2 in human pulp. Chung Hua Kou Chiang Hsueh Tsa Chih. 1999;34: 236-238. PMID: 11776916

84. Khabbaz MG, Anastasiadis PL, Sykaras SN. Determination of endotoxins in the vital pulp of human carious teeth: association with pulpal pain. Oral Surg Oral Med Oral Pathol Oral Radiol Endod. 2001;91: 587-593. PMID: 11346740

85. Nakanishi T, Shimizu H, Hosokawa Y, Matsuo T. An immunohistological study on cyclooxygenase-2 in human dental pulp. J Endod. 2001;27: 385-388. PMID: 11487130

86. Guven G, Altun C, Gunhan O, Gurbuz T, Basak F, Akbulut E, et al. Co-expression of cyclooxygenase-2 and vascular endothelial growth factor in inflamed human pulp: an immunohistochemical study. J Endod. 2007;33: 18-20. PMID: 17185121

87. Awawdeh L, Lundy FT, Shaw C, Lamey PJ, Linden GJ, Kennedy JG. Quantitative analysis of substance P, neurokinin A and calcitonin gene-related peptide in pulp tissue from painful and healthy human teeth. Int Endod J. 2002;35: 30-36. PMID: 11853236

88. Caviedes-Bucheli J, Lombana N, Azuero-Holguin MM, Munoz HR. Quantification of neuropeptides (calcitonin gene-related peptide, substance P, neurokinin A, neuropeptide Y and vasoactive intestinal polypeptide) expressed in healthy and inflamed human dental pulp. Int Endod J. 2006;39: 394-400. PMID: 16640639

89. Caviedes-Bucheli J, Camargo-Beltran C, Gomez-la-Rotta AM, Moreno SC, Abello GC, Gonzalez-Escobar JM. Expression of calcitonin gene-related peptide (CGRP) in irreversible acute pulpitis. J Endod. 2004;30: 201-204. PMID: 15085045

90. Caviedes-Bucheli J, Arenas N, Guiza O, Moncada NA, Moreno GC, Diaz E, et al. Calcitonin gene-related peptide receptor expression in healthy and inflamed human pulp tissue. Int Endod J. 2005;38: 712-717. PMID: 16164685

91. Artese L, Rubini C, Ferrero G, Fioroni M, Santinelli A, Piattelli A. Vascular endothelial growth factor (VEGF) expression in healthy and inflamed human dental pulps. J Endod. 2002;28: 20-23. PMID: 11806643

92. Caviedes-Bucheli J, Gutierrez-Guerra JE, Salazar F, Pichardo D, Moreno GC, Munoz HR. Substance P receptor expression in healthy and inflamed human pulp tissue. Int Endod J. 2007;40: 106-111. PMID: 17229115

93. Caviedes-Bucheli J, Moreno GC, Lopez MP, Bermeo-Noguera AM, Pacheco-Rodriguez G, Cuellar A, et al. Calcitonin gene-related peptide receptor expression in alternatively activated monocytes/macrophages during irreversible pulpitis. J Endod. 2008;34: 945-949. doi: 10.1016/j.joen.2008.05.011 PMID: 18634925

94. Zhong S, Zhang S, Bair E, Nares S, Khan AA. Differential expression of microRNAs in normal and inflamed human pulps. J Endod. 2012;38: 746-752. doi: 10.1016/j.joen.2012.02.020 PMID: 22595106

95. Dong Y, Lan W, Wu W, Huang Z, Zhao J, Peng L, et al. Increased expression of EphA7 in inflamed human dental pulp. J Endod. 2013;39: 223-227. doi: 10.1016/j.joen.2012.11.020 PMID: 23321235
